# Distinct multilevel misregulations of Parkin and PINK1 revealed in cell and animal models of TDP-43 proteinopathy

**DOI:** 10.1038/s41419-018-1022-y

**Published:** 2018-09-20

**Authors:** Xing Sun, Yongjia Duan, Caixia Qin, Jian-Chiuan Li, Gang Duan, Xue Deng, Jiangxia Ni, Xu Cao, Ke Xiang, Kuili Tian, Chun-Hong Chen, Ang Li, Yanshan Fang

**Affiliations:** 1 Interdisciplinary Research Center on Biology and Chemistry, Shanghai Institute of Organic Chemistry, Chinese Academy of Sciences, Shanghai, 201210 China; 2 University of Chinese Academy of Sciences, Beijing, 100049 China; 3 Guangdong-Hong Kong-Macau Institute of CNS Regeneration, Joint International Research Laboratory of CNS Regeneration Ministry of Education, Guangdong Medical Key Laboratory of Brain Function and Diseases, Jinan University, Guangzhou, 510632 China; 4 National Institute of Infectious Diseases and Vaccinology, National Health Research Institutes, Miaoli County, Taiwan

## Abstract

Parkin and PINK1 play an important role in mitochondrial quality control, whose malfunction may also be involved in the pathogenesis of amyotrophic lateral sclerosis (ALS). Excessive TDP-43 accumulation is a pathological hallmark of ALS and is associated with Parkin protein reduction in spinal cord neurons from sporadic ALS patients. In this study, we reveal that Parkin and PINK1 are differentially misregulated in TDP-43 proteinopathy at RNA and protein levels. Using knock-in flies, mouse primary neurons, and TDP-43^Q331K^ transgenic mice, we further unveil that TDP-43 downregulates *Parkin* mRNA, which involves an unidentified, intron-independent mechanism and requires the RNA-binding and the protein–protein interaction functions of TDP-43. Unlike *Parkin*, TDP-43 does not regulate *PINK1* at an RNA level. Instead, excess of TDP-43 causes cytosolic accumulation of cleaved PINK1 due to impaired proteasomal activity, leading to compromised mitochondrial functions. Consistent with the alterations at the molecular and cellular levels, we show that transgenic upregulation of Parkin but downregulation of PINK1 suppresses TDP-43-induced degenerative phenotypes in a *Drosophila* model of ALS. Together, these findings highlight the challenge associated with the heterogeneity and complexity of ALS pathogenesis, while pointing to Parkin–PINK1 as a common pathway that may be differentially misregulated in TDP-43 proteinopathy.

## Introduction

Amyotrophic lateral sclerosis (ALS) is an adult-onset neurodegenerative disease characterized by progressive motor neuron loss, leading to muscle weakness and wasting. ALS is incurable and the patients usually die within 2–5 years after diagnosis. The majority (>90%) of ALS cases are sporadic (sALS) with unknown causes, whereas mutations in genes such as *SOD1*, *C9orf72*, *FUS*, and *TARDBP* are reported to cause familial ALS that accounts for the remaining 10%^1,2^. Ubiquitin-positive cytoplasmic inclusions containing transactive response DNA-binding protein 43 kDa (TDP-43, encoded by *TARDBP*) in the brain and the spinal cord of patients are a main pathological hallmark of ALS^3^. Moreover, TDP-43-positive protein inclusions are found in a large spectrum of neurodegenerative disorders, including frontotemporal lobar degeneration, Alzheimer’s disease, dementia with Lewy bodies, polyglutamine diseases, and others^4–8^, which are collectively known as TDP-43 proteinopathy.

In physiological conditions, TDP-43 protein is predominantly localized to the nucleus. It belongs to the heterogeneous ribonucleoprotein family and plays an important role in regulating gene transcription, RNA processing, transport, and stability, as well as the formation of stress granules. In disease conditions, ubiquitinated TDP-43 accumulates in the cytoplasm. The mislocalization and aberrant aggregation of TDP-43 cause dysfunction of various aspects of RNA metabolism as well as protein homeostasis, eventually leading to motor neuron degeneration^9–11^. With the recent advancement of the next-generation sequencing, the RNA targets as well as the common principles of TDP-43-mediated RNA regulations are emerging^12–15^.

The long intron-containing pre-mRNA of* Parkin* is one of the reported targets of TDP-43^13^. Parkin is an E3 ubiquitin ligase involved in the clearance of damaged mitochondria via autophagy (termed “mitophagy”), which partners with PTEN-induced putative kinase 1 (PINK1) to execute the mitochondrial quality control function. PINK1 is a serine/threonine kinase, which after translation is continuously transported into mitochondria, cleaved, and released to the cytosol for proteasome-mediated degradation^16,17^. When mitochondria are damaged, PINK1 cannot be effectively cleaved and is subsequently anchored on the mitochondrial outer membrane, which in turn recruits Parkin to mitochondria and induces mitophagy^18^. Mutations in *Parkin* and *PINK1* genes are linked to autosomal recessive early-onset Parkinson’s disease (PD)^19,20^.

Increasing evidence points to mitochondrial dysfunction as a common pathogenic factor in ALS^21,22^. Interestingly, in the spinal cord autopsy samples from sALS patients, neurons with TDP-43 protein inclusions have reduced Parkin protein levels^12^. In this study, we investigate whether Parkin and PINK1 are involved in TDP-43-induced neurodegeneration. We find that Parkin and PINK1 are differentially misregulated by TDP-43 at the RNA and protein levels. Consistently, genetic manipulations of Parkin or PINK1 exhibit the opposing modifying effects in a *Drosophila* model of TDP-43 proteinopathy. Collectively, we propose that distinct multilevel misregulations of Parkin and PINK1 contribute to the pathogenesis of TDP-43 proteinopathy.

## Results

### TDP-43 overexpression selectively decreases *Parkin* but not *PINK1* mRNA levels

**Fig. 1:**
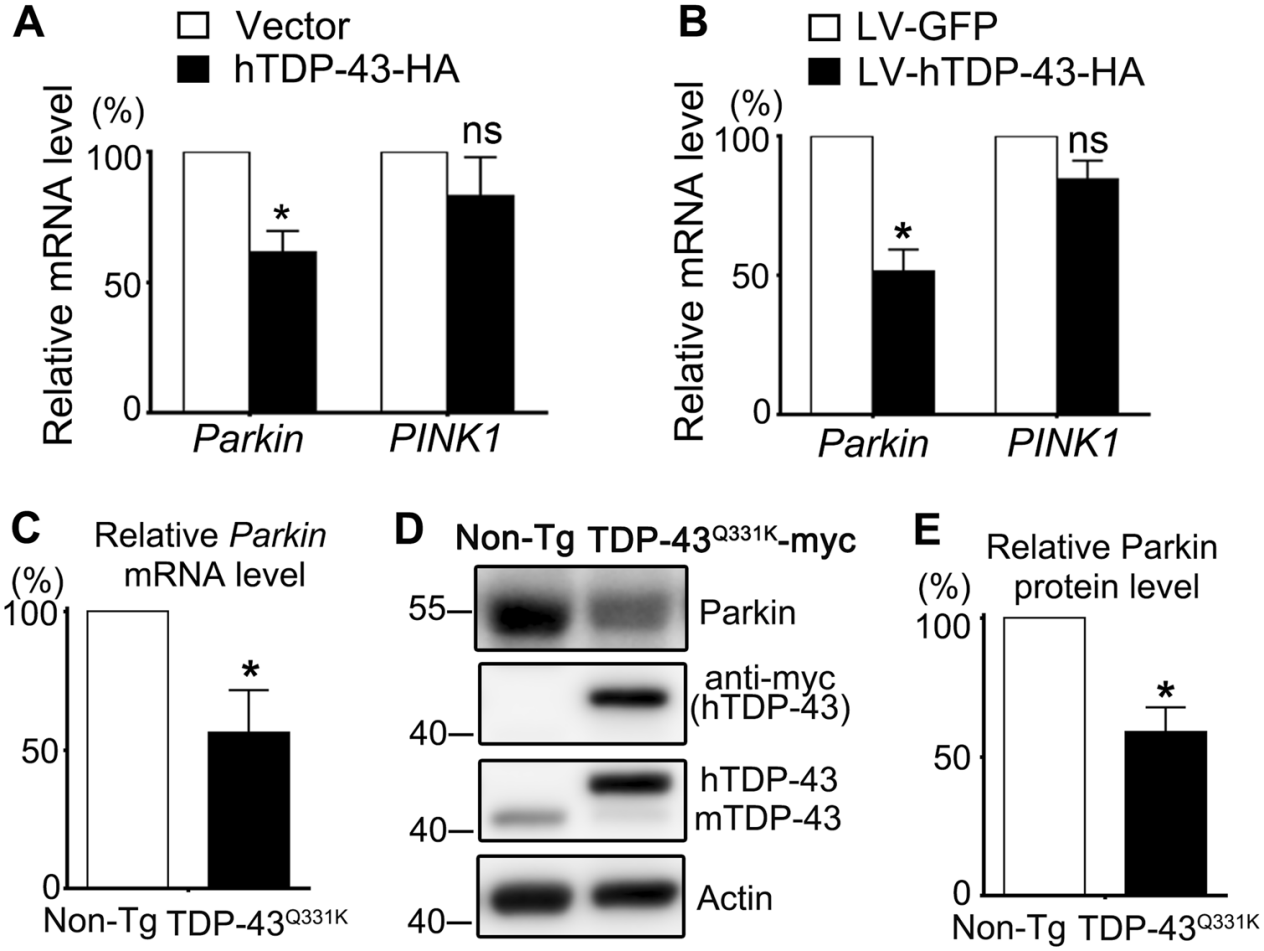
*Parkin* mRNA and protein levels are decreased in mammalian cell and primary neuron models of TDP-43 proteinopathy. **a**, **b** qPCR analysis of the mRNA levels of *Parkin* and *PINK1* in human 293T cells (**a**) and primary mouse neurons infected with wild-type hTDP-43 (**b**). **c**–**e** The endogenous mouse *Parkin* mRNA (**c**) and protein (**d**) levels of the cortical neurons derived from the hTDP-43^Q331K^ mice are decreased compared to the non-transgenic sibling controls. Non-Tg non-transgenics, hTDP-43 transgenic human TDP-43, mTDP-43 endogenous mouse TDP-43. Parkin protein levels are normalized to Actin and plotted as percentage to controls in (**e**). Data are shown as means ± SEM; *n* = 3–5; *p < 0.05; ns not significant; Student’s *t*-test

Previous studies showed that TDP-43 regulated *Parkin* pre-mRNA and TDP-43 loss of function (LOF) reduced *Parkin* mRNA levels in mouse brains^12,13^. Since TDP-43-induced neurodegeneration involves both LOF and gain-of-function (GOF) mechanisms^23^, in this study we investigated whether TDP-43 GOF affected *Parkin* and *PINK1*, starting with overexpressing human TDP-43 (hTDP-43) in mammalian systems. We examined the mRNA levels of *Parkin* and *PINK1* in human 293T cells transfected with hTDP-43-HA and mouse primary neurons infected with lentivirus to express hTDP-43-HA (Fig. S1A). In both 293T cells (Fig. 1a) and primary mouse neurons (Fig. 1b), TDP-43 overexpression (OE) caused a significant reduction of *Parkin* mRNA levels compared to each of the control groups. However, in neither 293T cells (Fig. 1a) nor mouse neurons (Fig. 1b) did TDP-43 OE significantly alter *PINK1* mRNA levels. Further, we found that in neurons derived from the transgenic mice expressing mutant hTDP-43^Q331K^^24^ (Fig. S1B), *Parkin* mRNA and protein levels were also significantly decreased (Fig. 1c–e). Of note, the endogenous mouse TDP-43 protein level was decreased in hTDP-43^Q331K^-derived neurons, which was likely due to the inhibition of its own transcription by hTDP-43 OE as reported previously^24^. The total expression levels of both endogenous and exogenous TDP-43 proteins were examined in Fig. S1C–D.

**Fig. 2:**
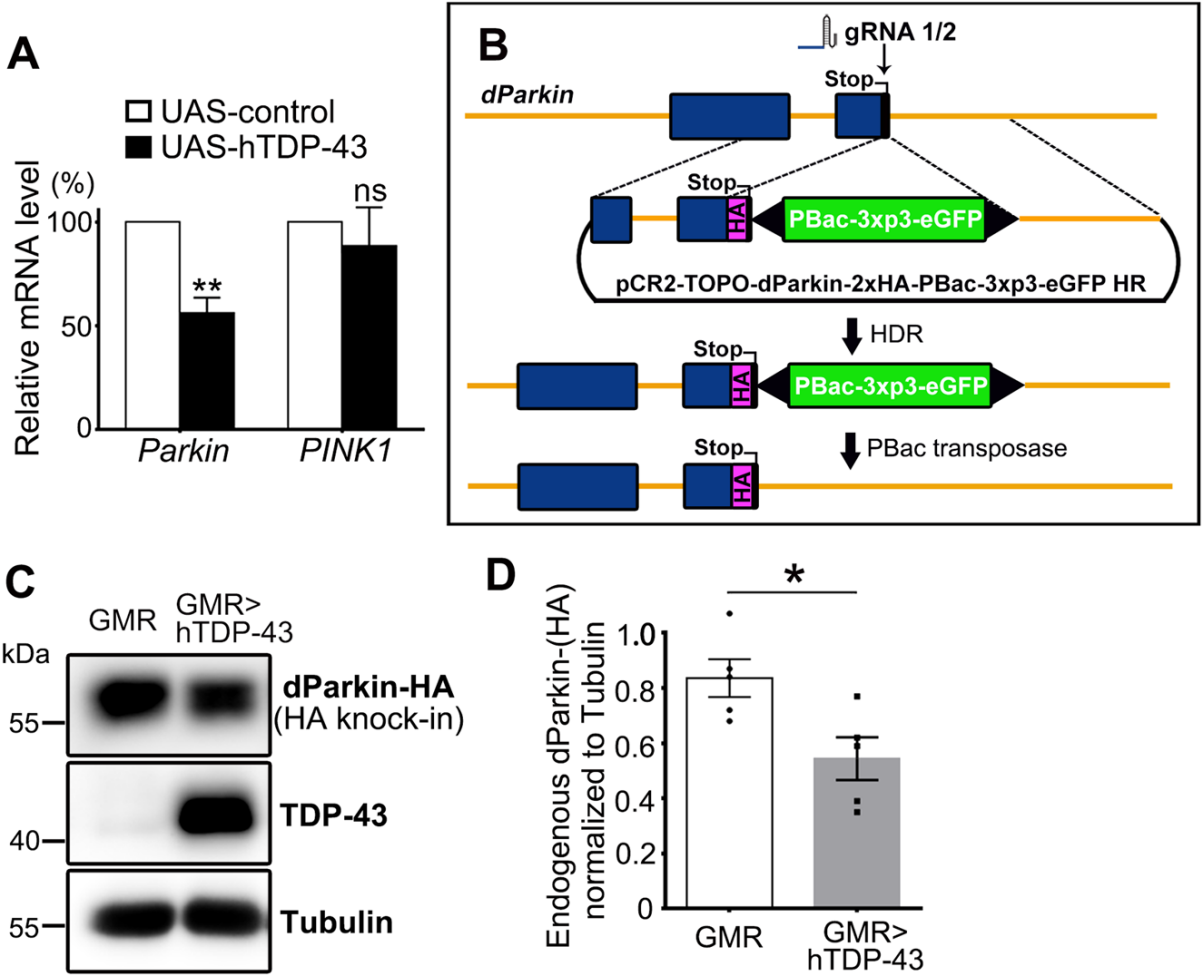
*Parkin* mRNA and protein levels are reduced in the TDP-43 flies. **a** qPCR analysis of the mRNA levels of *Parkin* and *PINK1* in the hTDP-43 fly heads (with a GMR-Gal4 driver). The mRNA levels of *Parkin* and *PINK1* are normalized to *actin* and shown as average percentages to that of the control group. **b** A simplified overview of the process to generate the *dParkin*-(HA) knock-in flies using the transgenic Cas9-gRNA system (graph adapted from the flyCRISPR website). A pCR2-TOPO-dParkin-2xHA-PBac-3xp3-eGFP HR donor vector is generated to insert a 2xHA tag into the fly genome at the C terminus of the *dParkin* gene by homology-directed repair (HDR). The successful knock-ins are identified by the GFP selection marker, which is subsequently removed by PBac transposase, leaving only the HA-tag and the stop codon in the target locus. **c**, **d** Western blot analysis of the endogenous Parkin protein levels measured by the knock-in HA-tag in the hTDP-43 flies or the driver only control (GMR) flies. The quantification is shown in **d**. Data are means ± SEM; *n* = 5; *p < 0.05, **p < 0.01; ns not significant; Student’s *t*-test

To confirm this effect in an in vivo system, we examined mRNA levels of *Parkin* and *PINK1* in the fly head of a previously established *Drosophila* ALS model of TDP-43^25,26^. Consistently, we detected an almost 50% reduction of the *Parkin* mRNA levels in the hTDP-43 fly heads (Fig. 2a). In contrast, TDP-43 OE did not significantly affect *PINK1* mRNA abundance (*p* = 0.565, Fig. 2a). Hence, the mRNA levels of *Parkin* but not *PINK1* were selectively reduced in multiple human cell, primary mouse neuron and in vivo fly models of TDP-43 proteinopathy.

### Generation of the dParkin-(HA) knock-in fly and examination of TDP-43 OE on Parkin protein levels in vivo

Next, we sought to confirm the effect of TDP-43 OE on Parkin protein levels in the in vivo fly models. Unfortunately, none of the commercially available Parkin antibodies we have examined in this study worked in western blots with *Drosophila* Parkin protein (data not shown). To overcome this problem, we utilized the CRISPR-based gene editing and the transgenic Cas9-gRNA system^27,28^ to generate an HA knock-in (KI) fly in which an HA-tag was inserted to the C terminus of the endogenous *Drosophila Parkin* (*dParkin*) gene (Fig S2A). Briefly, we constructed an HA donor vector flanked with *dParkin* sequences (pCR2-TOPO-*dParkin*-2xHA-PBac-3xp3-eGFP) and two *dParkin*-stop codon guide RNA (gRNA) vectors (pBFv-U6.3-*dParkin*-stop gRNA-1 and gRNA-2). The HA donor and the *dParkin*-stop gRNA plasmids were mixed for micro-injection using a nanos-Cas9 founder line. The desired transformants were selected for subsequent balancing and background clearing, and were eventually established as a stable dParkin-(HA) KI line (Fig. 2b and Methods).

The 2xHA tag inserted to the C terminus of the endogenous *dParkin* gene allowed us to measure the endogenous Parkin protein levels by probing the HA-tag. Next, we crossed the dParkin-(HA) KI flies to hTDP-43 flies and examined the dParkin-HA protein levels by western blotting. Compared to the control group (GMR driver only), expression of hTDP-43 in the fly eyes caused a significant reduction of the endogenous Parkin protein levels (Fig. 2c, d). Together, both the mRNA and protein levels of *Parkin* were decreased in the *Drosophila* model of TDP-43 proteinopathy.

### TDP-43 can decrease the mRNA and protein levels of *Parkin* in the absence of intron/untranslated regions

Previous RNA-seq studies indicated that TDP-43 preferentially regulated pre-mRNAs with exceptionally long introns that are often more than 100 kb^12–15^. Human and mouse *Parkin* pre-mRNAs contained very long introns (Fig. S2B) and TDP-43 regulated their mRNA levels^12,13^. Intriguingly, we noticed that the *dParkin* gene contains only short introns (the longest <200 bp, Fig. S2A). This raised the possibility that in addition to the reported intron-mediated regulation, TDP-43 might also regulate *Parkin* mRNA levels by an intron-independent mechanism. To test this hypothesis, we generated the transient expression plasmid that contains only the coding region of the human *Parkin* gene (Flag-Parkin, Fig. S2C).

**Fig. 3:**
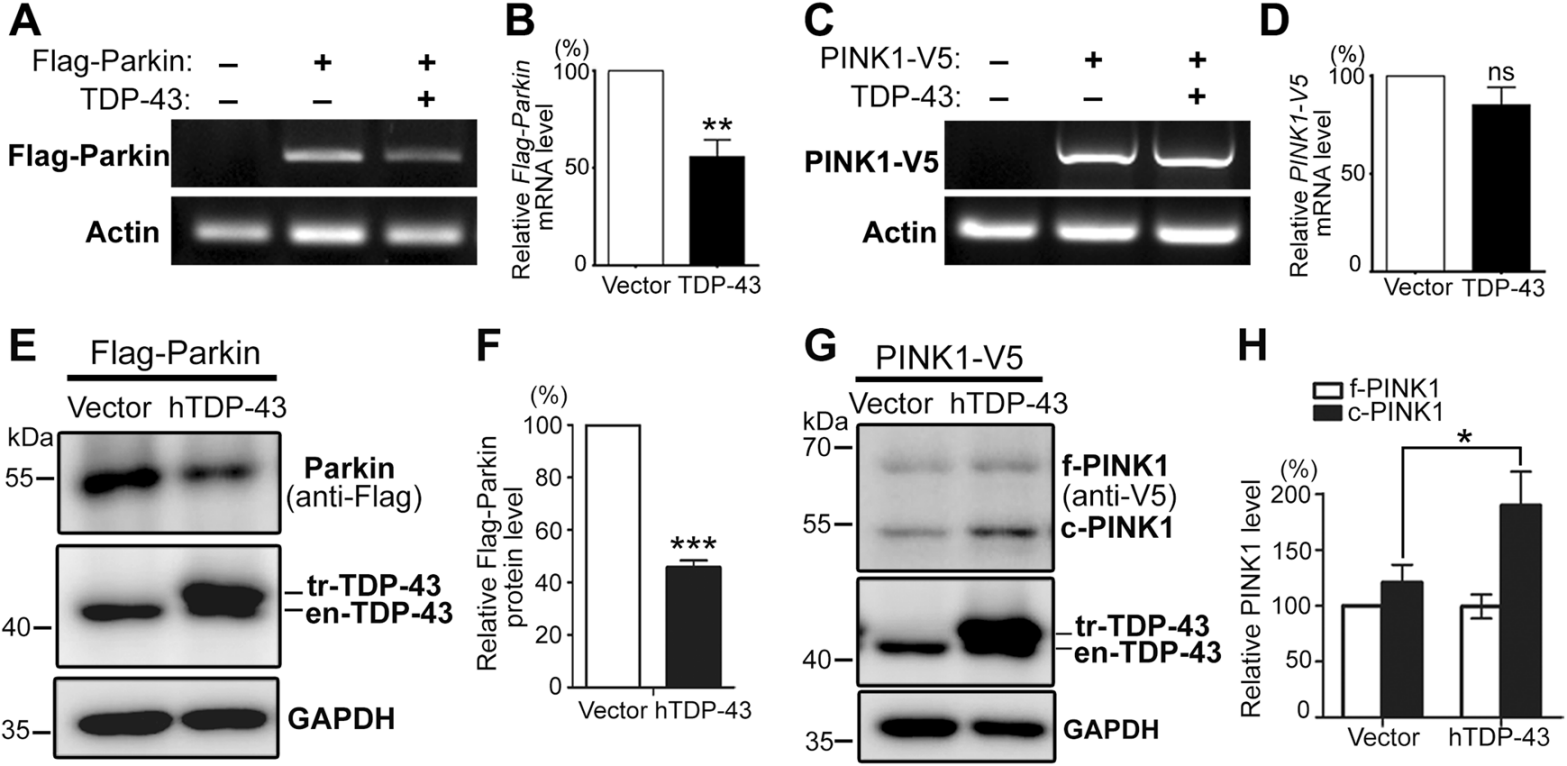
TDP-43 OE can reduce the mRNA and protein levels of *Parkin* in an intron-independent manner. **a**–**d** TDP-43 selectively reduces the mRNA levels of plasmid-expressed, intron-free *Parkin* but not *PINK1*. The mRNA levels of transiently transfected *Flag-Parkin* (**a**) or *PINK1-V5* (**c**) in 293T cells are evaluated by a semiquantitative RT-PCR assay, and quantified in **b** and **d**, respectively. The specific PCR primers (containing the Flag or V5 tag sequence) are designed to distinguish the plasmid mRNAs from endogenously expressed *Parkin* and *PINK1* mRNAs in 293T cells. **e**, **f** TDP-43 OE decreases the protein levels of Parkin but increases cleaved PINK1. Western blot analysis of protein levels of plasmid-expressed Flag-Parkin (**e**) or PINK1-V5 (**g**) in 293T cells, and quantified in **f** and **h**, respectively. All protein levels are normalized to GAPDH. en-TDP-43 endogenous human TDP-43 in 293T cells, tr-TDP-43 transfected hTDP-43-HA, f-PINK1 full-length PINK1, c-PINK1 cleaved PINK1. Data are means ± SEM; *n* = 3–5; *p < 0.05, **p < 0.01, ***p < 0.001; ns not significant; Student’s *t*-test

We then examined whether TDP-43 could downregulate the mRNA levels of the plasmid-expressed, intron-free human *Parkin*, in mammalian cells. An intron-free *PINK1* (PINK1-V5) constructed in the same expression plasmid was included as a control. The reverse transcription-PCR (RT-PCR) primers were designed to distinguish the Flag-tagged *Parkin* and V5-tagged *PINK1* from the endogenously expressed *Parkin* and *PINK1* mRNA in 293T cells (see Methods). As shown in Fig. 3a, b, the mRNA levels of *Flag-Parkin* were reduced by about 50% in TDP-43-overexpressing cells, whereas *PINK1-V5* mRNA was not significantly changed (Fig. 3c, d). Consistently, the protein levels of the exogenous, intron-free Flag-Parkin were also drastically decreased by TDP-43 OE (Fig. 3e, f). In contrast, the protein levels of full-length (FL) PINK1 were not decreased, whereas cleaved PINK1 was increased (Fig. 3g, h; to be addressed later in this study). These data indicated that TDP-43 could regulate *Parkin* mRNA and protein levels in the absence of introns in mammalian cells, suggesting that TDP-43-mediated reduction of Parkin levels in sALS patients might involve both intron-based and intron-independent regulations of *Parkin* mRNA. Furthermore, since *Parkin* and *PINK1* were subcloned into the same vector with the same backbone and promoter (see Methods), it was unlikely that the selective downregulation of plasmid-expressed, intron-free *Parkin* was due to a general effect of TDP-43 on transcription or the untranslated regions (UTRs) of the expression plasmid.

### Downregulation of intron-free *Parkin* requires both the RRM1 and glycine-rich domains of TDP-43

**Fig. 4:**
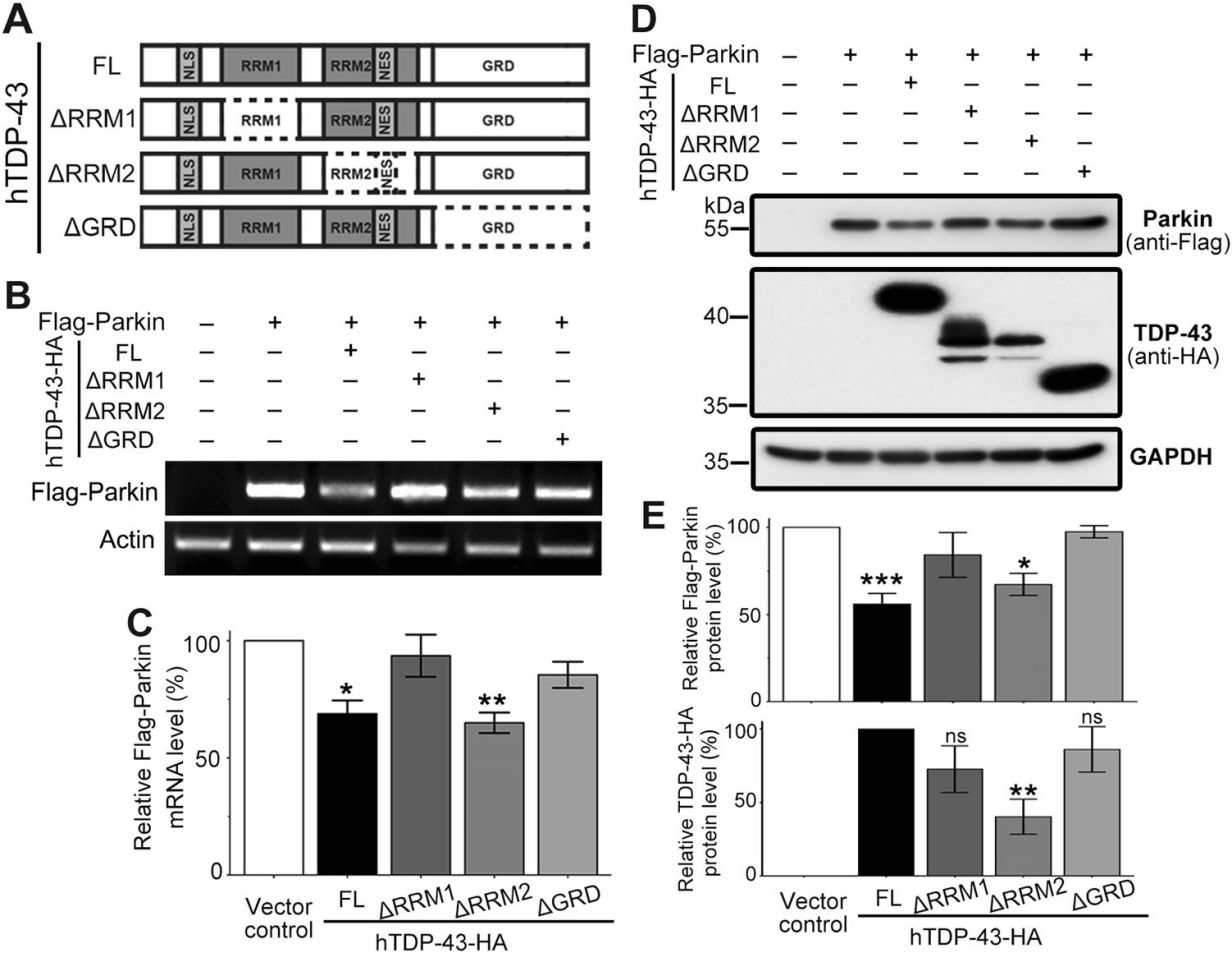
The RRM1 and GRD domains of TDP-43 are required for downregulation of intron-free Parkin. **a** A schematic graph showing the major TDP-43 functional domains and the truncated TDP-43 proteins examined in this study. NLS nuclear localization signal, NES nuclear export signal, RRM RNA recognition motif, GRD glycine-rich domain. **b**–**e** Semiquantitative RT-PCR and western blotting analyses of mRNA (**b**) or protein levels (**d**) of plasmid-expressed, intron-free *Flag*-*Parkin* co-expressed with full-length (FL) or truncated hTDP-43-HA as indicated. The PCR primers (containing the Flag tag sequence) are designed to only amplify the plasmid-expressed *Flag-Parkin* but not the endogenous *Parkin*, confirmed by the negative control. The quantifications of **b** and **d** were shown in **c** and **e**, respectively. The relative mRNA and protein levels of Flag-Parkin in the vector control group and the protein level of TDP-43 in the hTDP-43-FL group are set to 100%. Data are means ± SEM; *n* = of 3–5; *p < 0.05, **p < 0.01, ***p < 0.001; ns not significant; one-way ANOVA

TDP-43 is a RNA-binding protein that consists of a nuclear localization signal and a nuclear export signal, two RNA recognition motif (RRM) motifs involved in DNA and RNA binding, and a glycine-rich domain (GRD) domain at the C terminus that mediates protein–protein interaction with other members of the heterogeneous nuclear RNP protein family^9,10^. To understand what motif(s) are required and how TDP-43 regulates Parkin in the absence of any intron or gene-specific UTR, we generated the plasmids to express truncated TDP-43 protein: hTDP-43-HA-ΔRRM1, -ΔRRM2, and -ΔGRD (Fig. 4a). We found that hTDP-43-ΔRRM1 and hTDP-43-ΔGRD were unable to significantly downregulate the intron-free *Parkin* mRNA levels, whereas hTDP-43-ΔRRM2 showed a similar reduction of *Parkin* mRNA to that of the FL TDP-43 (Fig. 4b, c). Further, we examined the effects of FL and truncated TDP-43 on the protein levels of intron-free *Parkin*. Consistently, hTDP-43-ΔRRM1 and hTDP-43-ΔGRD could not downregulate the protein levels of Flag-Parkin, whereas hTDP-43-ΔRRM2 significantly decreased Flag-Parkin protein levels, and this was despite the fact that hTDP-43-ΔRRM2 was expressed at a lower level (Fig. 4d, e). These results suggest that the post-transcriptional, intron/UTR-independent downregulation of *Parkin* requires both the RNA-binding and protein–protein interaction functions of TDP-43.

### Increase of TDP-43 causes cytosolic accumulation of cleaved PINK1 that forms insoluble aggregates in the cell

**Fig. 5:**
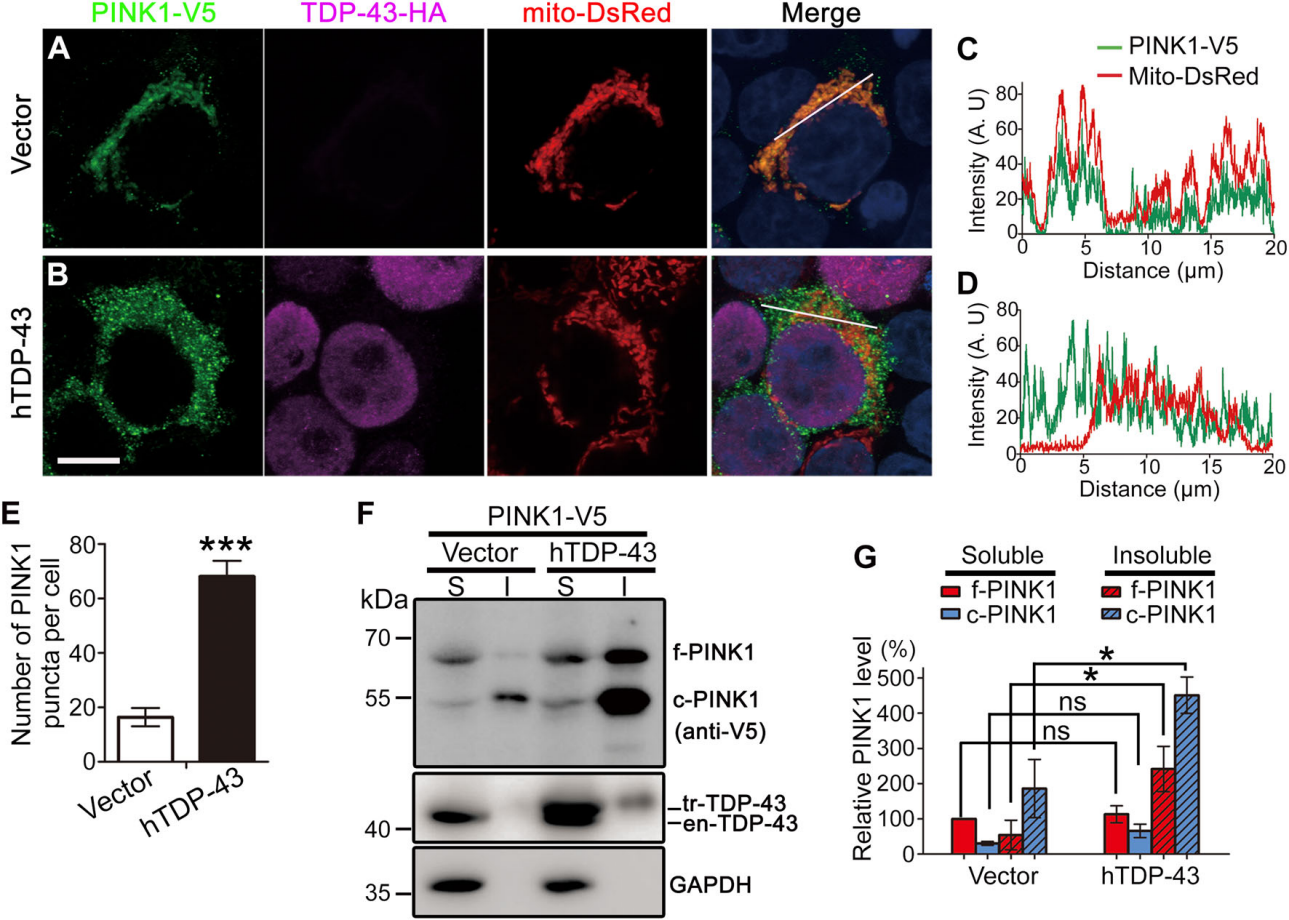
TDP-43 OE promotes cytosolic accumulation of cleaved PINK1 that forms insoluble aggregates. **a**, **d** 293T cells transiently transfected with PINK1-V5 in the absence (vector control) (**a**) or presence of co-transfection of hTDP-43-HA (**b**). Representative confocal images show cells immunostained for PINK1 (V5) and TDP-43 (HA). Mitochondria are labeled by mito-DsRed and a merge of all channels is shown with the nucleus stained by DAPI in blue. The co-localization of PINK1 with mitochondria (mito-DsRed) is evaluated by the line scanning analysis in **c** and **d**. **e** The number of protein puncta is counted using the “Analyze Particles” module of ImageJ and shown as average numbers of puncta per cell. Data are means ± SEM of ~100 cells per group; ***p < 0.001. Scale bar: 10 μm. **f**, **g** 293T cells transfected of PINK1-V5 together with empty vector (control) or hTDP-43 are lysed and fractionated. The RIPA-soluble and -insoluble (resolved in 9 M urea) fractions are analyzed by western blotting (**f**) and quantified in **g**. S soluble fraction, I insoluble fraction, en-TDP-43 endogenous TDP-43, tr-TDP-43 transfected hTDP-43-HA, f-PINK1 full-length PINK1, c-PINK1 cleaved PINK1. All protein levels are normalized to GAPDH. The relative protein level of soluble full-length PINK1 in the control group (vector) is set to 100%. Data are means ± SEM; *n* = 3; *p < 0.05; Student’s *t*-test; ns not significant

We noticed that although TDP-43 OE did not significantly change *PINK1* mRNA or FL PINK1 protein levels (~64 kDa), there was a remarkable increase of cleaved PINK1 (~52 kDa^16,29^) in the western blots (Fig. 3g, h). Damaged mitochondria accumulate PINK1 on the outer membrane, which in turn recruits Parkin and induces mitophagy^18^. To determine if the TDP-43-induced increase of cleaved PINK1 was due to mitochondrial accumulation of PINK1, we examined the subcellular localization of PINK1 by immunocytochemistry. Since the expression of endogenous PINK1 protein was too low to be reproducibly detected in 293T cells, we transfected the cells with PINK1-V5 and immunostained for the V5 tag. In the control cells, PINK1-V5 was predominantly co-localized with mito-DsRed (Fig. 5a), indicating that PINK1 was mostly localized to mitochondria under normal conditions. With TDP-43 OE, PINK1-V5 was no longer specifically localized to mitochondria but spread out in the cytoplasm (Fig. 5b). The reduced mitochondrial localization was further demonstrated by the co-localization analysis of PINK1-V5 and mito-DsRed (Fig. 5c, d).

In addition to the altered subcellular distribution of PINK1, another striking phenomenon we observed was that PINK1 formed massive puncta in the cells co-transfected with hTDP-43 (Fig. 5b, e). To determine if the PINK1 puncta were protein aggregates, we assessed the solubility of PINK1 by fractionation and western blot. In control cells, the FL PINK1 was mainly in the soluble fraction and the cleaved PINK1 was in the insoluble fraction. With TDP-43 OE, there was a robust increase of both the FL and cleaved PINK1 levels in the insoluble fraction, of which the cleaved PINK1 was more dramatically increased (Fig. 5f, g). In contrast, TDP-43 did not significantly alter Parkin subcellular distribution or form insoluble aggregates (Fig. S3). Together, we concluded that TDP-43 OE led to a cytosolic accumulation of cleaved PINK1 that formed insoluble protein aggregates.

### PINK1 proteostasis is sensitive to TDP-43-induced proteasomal activity impairment

In normal cells, cleaved PINK1 is released from mitochondria and undergoes rapid degradation in the cytoplasm via the ubiquitin proteasome system (UPS)^17^. As such, PINK1 protein usually is undetectable or at very low levels in healthy cells. The observation of TDP-43-induced cytosolic accumulation of cleaved PINK1 (Fig. 5) strongly suggested that the function of UPS might be impaired in TDP-43 proteinopathy. To test this possibility, we measured the proteasomal activity of the cells using an in vitro fluorogenic peptide cleavage assay. Compared to the control cells, there was a small but significant reduction of proteasomal activity of the cells transfected with hTDP-43 (Fig. S4A). Considering that TDP-43-mediated neurodegeneration in diseases usually develops over years, we think a small decrease rather than an abrupt inhibtion of the proteasomal function may be more pathophysiologically relevent to the disease condition.

Since the reduction of proteasomal activity by TDP-43 OE indicated by the in vitro assay was rather moderate, we asked if it was sufficient to significantly disturb the proteostasis of PINK1. To address this question, we treated the 293T cells with the proteasome inhibitor MG-132 at a series of concentrations, ranging from 5 nM to 5 μM for 3 h (Fig. S4B). The concentration of 50 nM generated a mild inhibition that is similar to the effect of hTDP-43 OE in 293T cells (Fig. S4A). We then treated the cells with MG-132 using this condition. Similar to hTDP-43 OE, no increase of Parkin intensity or Parkin protein aggregate was observed at this condition (Fig. S4C–D, G). In contrast, the same treatment of MG-132 inhibition caused a robust increase of PINK1 overall intensity and a massive accumulation of PINK1 aggregates in the cytoplasm (Fig. S4E–F, H). This result was consistent with the effects of TDP-43 OE on Parkin (Fig. S3) and PINK1 (Fig. 5), indicating that the turnover of PINK1 protein was extremely sensitive to alterations of proteasomal activity. Hence, TDP-43-induced mild impairment of proteasomal activity selectively hindered the turnover of proteasome function-sensitive proteins such as PINK1, but not other less labile proteins such as Parkin. This gave rise to the distinct effects that TDP-43 OE downregulated Parkin levels but led to a cytosolic accumulation of PINK1. Thus, in addition to intron-based regulation of *Parkin* pre-mRNA^12,13^ and intron-independent regulation of *Parkin* mRNA (Figs. 3 and 4), a third level of misregulation was on PINK1 protein turnover due to impaired proteasomal activity in TDP-43 proteinopathy.

### Endogenous PINK1 protein accumulates in TDP-43^Q331K^ mice

**Fig. 6:**
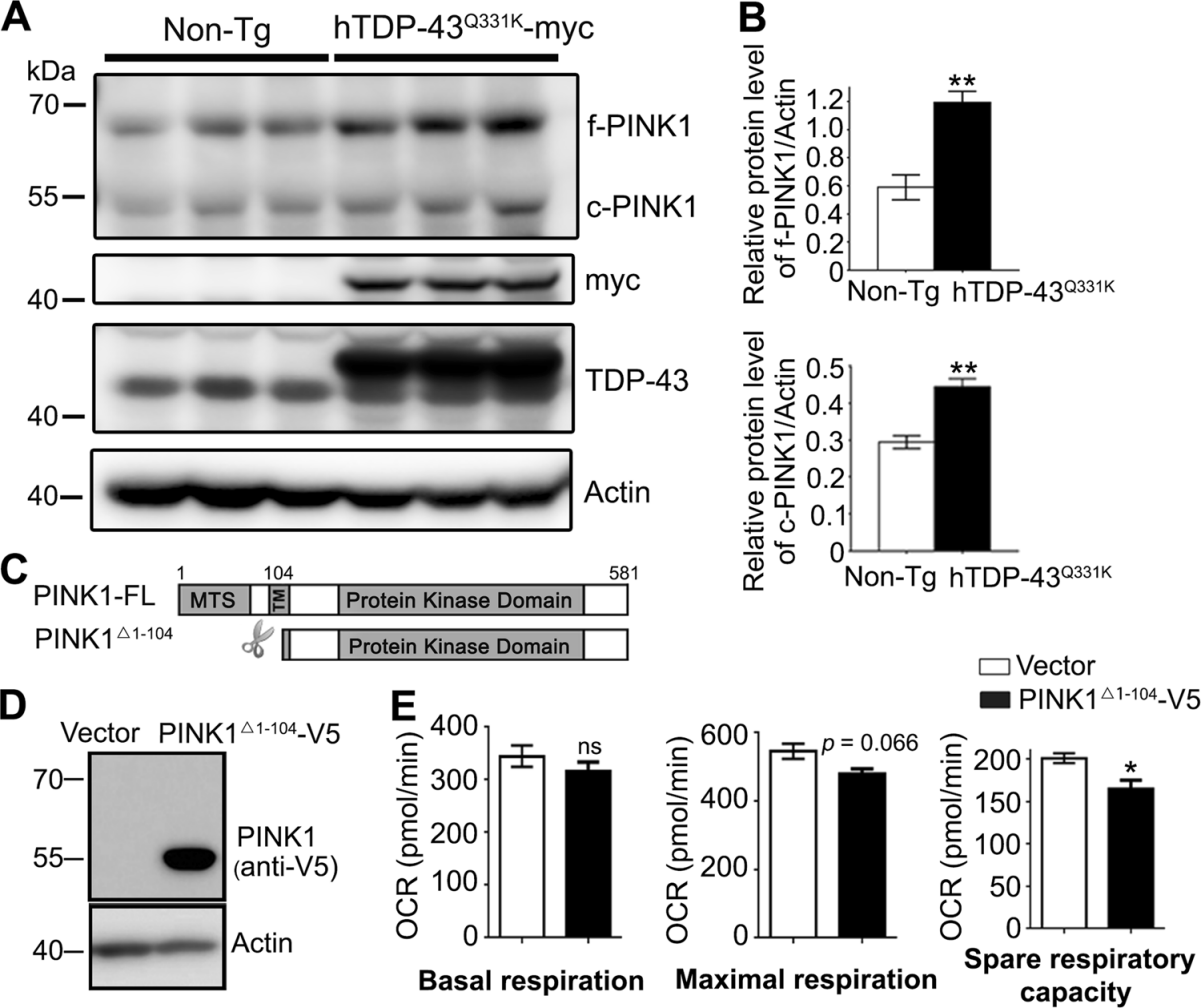
Accumulation of PINK1 proteins in TDP-43 mice and compromised mitochondrial functions with cleaved PINK1. **a**, **b** The endogenous PINK1 protein levels in the motor cortex of 8-month-old hTDP-43^Q331K^ mice or age-matched non-transgenic controls are analyzed by western blotting in **a** and quantified in **b**. Three mice of each genotype are tested. f-PINK1 full-length PINK1, c-PINK1 cleaved PINK1. All protein levels are normalized to actin. The relative expression levels of total TDP-43 proteins were examined in Fig. S1E. **c** A schematic drawing of the full-length PINK1-FL and truncated PINK1Δ^1-104^ after a cleavage at the amino acid 104. MTS mitochondrial targeting sequence, TM transmembrane domain. **d** A representative western blot confirming the PINK1Δ^1-104^-V5 expression (~53 kDa) in 293T cells. **e** The mitochondrial respiration functions in cells transfected with cleaved PINK1 are evaluated by the Seahorse XF Cell Mito Stress Test. The OCRs (pmol/min) of the basal, maximal respiration, and spare respiratory capacity are shown as means ± SEM; *n* = 3; *p < 0.05 and **p < 0.01; ns not significant; Student’s *t*-test

To confirm if endogenous PINK1 was similarly misregulated by TDP-43 in an in vivo mammalian model, we examined the PINK1 protein levels in the motor cortex of the 8-month-old TDP-43^Q331K^ mice. Consistently, we observed a remarkable increase of both FL and cleaved PINK1 proteins compared to the non-transgenic sibling control mice (Fig. 6a, b). As mentioned earlier, PINK1 protein is usually rapidly turned over and has a very low basal level in normal cells^17^. The accumulation of PINK1 protein in TDP-43 neurons is likely to be deleterious. Consistent with this idea, we observed that neuronal upregulation of *PINK1* reduced the *Drosophila* lifespan by ~6.9% (Fig. S5A–B).

### Increase of cleaved PINK1 reduces mitochondrial functions

To further elucidate the cellular consequence of PINK1 accumulation, especially the increase of cleaved PINK1, we generated the plasmid to express truncated PINK1 (PINK1^Δ1-104^-V5, Fig. 6c, d) that mimicked the protein product after the FL PINK1 was cleaved^17^. We used a Seahorse XFe96 Analyzer together with the Cell Mito Stress Test to measure the oxygen consumption rate (OCR) of the 293T cells transfected with PINK1^Δ1-104^-V5 or vector. The basal mitochondrial respiration occurred similarly, whereas the spare respiratory capacity was significantly reduced in PINK1^Δ1-104^-V5 cells (Fig. 6e); the maximal respiration also showed a trend to decrease although not statistically significant (*p* = 0.066; Fig. 6e). The data suggest that under normal conditions, cells with accumulation of cleaved PINK1 may function normally. However, under stress, these cells can exhibit reduced mitochondrial activity, which over years in aging might promote the pathogenesis of neurodegeneration in disease.

### Upregulation of *Parkin*, whereas downregulation of *PINK1* improves the degenerative phenotypes of TDP-43 flies

**Fig. 7:**
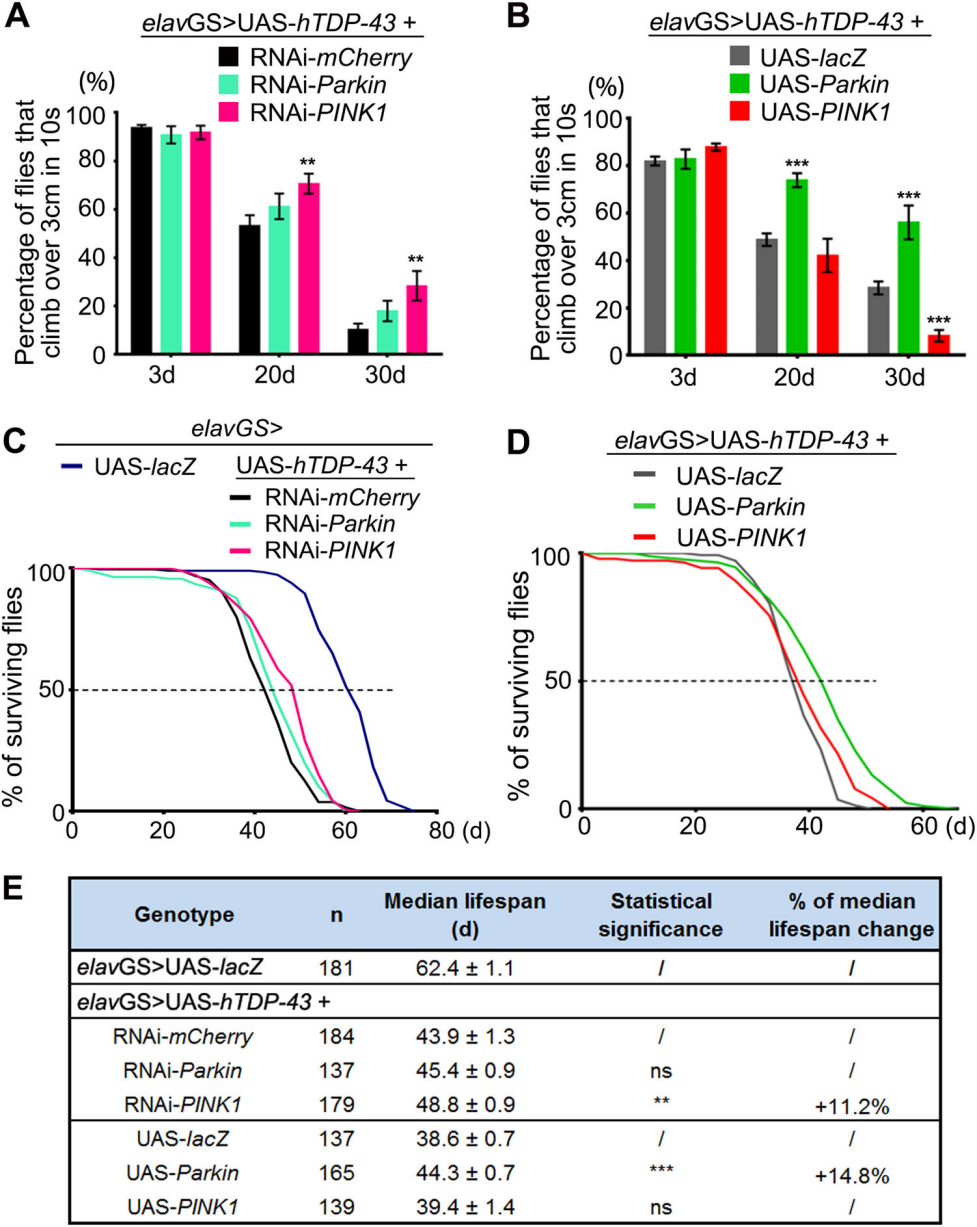
*Parkin* and *PINK1* show distinct modifying effects in a *Drosophila* model of TDP-43 proteinopathy. **a**, **b** The effect of KD (**a**) or OE (**b**) of *Parkin* or *PINK1* on hTDP-43 flies in climbing assays. RNAi-*mCherry* is used as a control for RNAi-*Parkin* and RNAi-*PINK1*, and UAS-*lacZ* is a control for UAS-*Parkin* and UAS-*PINK1*. Transgenic expression is restricted to the adult neurons using an *elav*GS driver (induced with RU486, 80 μg/ml, starting from day 1 of the adulthood). The locomotive capability is evaluated as the average percentage of flies that climb over 3 cm within 10 s. Data are shown as mean ± SEM, *n* = 20 flies/vial and 5 vials for each group. **c**–**e** *PINK1* KD or *Parkin* OE in the adult neurons significantly extends the lifespan of hTDP-43 flies. The lifespan assay of TDP-43 flies with adult-onset neuronal KD (**c**) or OE (**d**) of *Parkin* or *PINK1*. *elav*GS > UAS-*lacZ* is used as a non-disease UAS transgene control for UAS-hTDP-43. **e** Summary of the median lifespans, shown as mean ± SEM; *n*, the number of flies tested for each genotype is indicated; **p < 0.01, ***p < 0.001; ns not significant; Student’s *t*-test

After revealing the multilevel misregulation of Parkin and PINK1 by TDP-43 OE at the cellular level, we were keen to know whether their misregulations contribute to ALS pathogenesis and whether restoring *Parkin* and *PINK1* normal expression levels offers a means to ameliorate TDP-43-mediated neurodegeneration at the animal level. To answer these questions, we utilized an inducible *elav*-GeneSwich (*elav*GS) driver^59^ to express hTDP-43 in adult fly neurons only (induced after eclosion of the adult fly). Expression of hTDP-43 in adult fly neurons induced an age-dependent decline of climbing capability (see the control groups of RNAi-*mCherry* and UAS-*lacZ* in Fig. 7a, b). RNAi knockdown (KD) of *PINK1* but not *Parkin* significantly delayed the age-dependent climbing deficits of hTDP-43 flies (D20 and D30, Fig. 7a), in spite of a more efficient KD of *Parkin* than that of *PINK1* by the RNAi transgenes (Fig. S5C–D). On the contrary, *Parkin* OE provided remarkable suppression (D20 and D30, Fig. 7b), whereas *PINK1* OE enhanced the age-dependent climbing deficits of hTDP-43 flies (D30, Fig. 7b). The KD efficiency and OE levels of the UAS transgenes are confirmed in Figure S5. Moreover, KD or OE of *Parkin* in fly neurons did not significantly change the protein levels or subcellular distribution of hTDP-43; nor did the genetic manipulations of *PINK1* (Fig. S6). These data confirmed that the modifying effect of *Parkin* and *PINK1* in the hTDP-43 flies was not due to an alteration of the TDP-43 protein per se.

Next, we evaluated the effects of manipulating *Parkin* and *PINK1* expression levels on modifying the longevity of hTDP-43 flies. As shown in the lifespan experiments in Fig. 7c–e, the median lifespan of hTDP-43 flies was significantly increased by 11.2% with downregulation of *PINK1* and by 14.8% with upregulation of *Parkin* in adult fly neurons. Considering that TDP-43 is a DNA/RNA-binding protein and excessive TDP-43 impairs proteasomal activity (Fig. S4), it is likely that there are other targets whose mRNA abundance and protein turnover are also affected. Thus, it is unsurprising that correcting the misregulations of *Parkin* or *PINK1* only partially rescued the phenotypes of hTDP-43 flies. In an earlier study, we have shown that reduction of *PINK1* levels in adult neurons of wild-type flies did not have a dramatic effect on aging^30^. And, although ubiquitous *Parkin* OE extended *Drosophila* lifespan^31^, neuronal OE of *Parkin* showed minimal^31^ or no statistically significant increase of longevity (Fig. S5A–B). Thus, the improvement of TDP-43 flies’ survival was unlikely a generic effect of Parkin OE or PINK1 KD on aging. Rather, the results suggested a specific involvement of Parkin and PINK1 in TDP-43 proteinopathy.

## Discussion

Mitochondrial dysfunction has been linked to the pathogenesis of ALS^32–36^. *Parkin* and *PINK1* are both important genes involved in mitochondrial quality control, and previous sequencing studies identified *Parkin* pre-mRNA as a RNA target of TDP-43^12,13^. Interestingly, *Parkin* mRNA levels were decreased in the brain of TDP-43 KD mice; whereas in sALS patient cells, Parkin protein level decrease was associated with excessive TDP-43 accumulation^12^. This is likely because TDP-43-mediated neurodegeneration involves both LOF and GOF mechanisms^23^. In this study, we focus on how TDP-43 GOF affects *Parkin* and *PINK1*, and use multiple cell and animal models of TDP-43 proteinopathy to demonstrate that they are differentially misregulated at RNA and protein levels by TDP-43 OE.

In all models tested in this study, we observe a significant reduction of *Parkin* mRNA and protein levels (Figs. 1 and 2). Consistently, the increase of *Parkin* suppresses the degenerative phenotypes of hTDP-43 flies (Fig. 7) and reduces neuronal cell death in the motor cortex of rat TDP-43 model^37^. We speculate that the TDP-43 KD-induced *Parkin* mRNA reduction may be due to loss of TDP-43 binding and protection of the *Parkin* pre-mRNA^12,13^, whereas TDP-43 OE-induced *Parkin* decrease may result from abnormal binding and misregulation of mature *Parkin* mRNA (Figs. 3 and 4) or other mechanisms to be discovered. Nevertheless, such seemingly contradictory effects are also observed with TDP-43 in regulating alternative splicing. Taking the TDP-43 targets *Kcnd3* and *Ahi1* as an example, both KD and OE of TDP-43 cause aberrant alternative splicing of them in the same direction^24^.

As a RNA-binding protein, TDP-43 regulates various aspects of RNA metabolism, including the sustenance of long intron-containing pre-mRNA, alternative splicing, 3′UTR-mediated mRNA transport or stability, and regulation of long noncoding RNAs^2,12,13,15^. All of these regulations appear to require noncoding sequences such as introns and 3′UTRs. One intriguing finding of this study is that TDP-43 not only regulates the mRNA of long intron-containing human and mouse *Parkin* but also reduces the mRNA levels of *Parkin* transcribed from the *dParkin* gene with only very short introns. We further exemplify that in the absence of any intron or UTR, plasmid-expressed *hParkin* can still be downregulated by TDP-43 (Fig. 3), which suggests an additional, intron-independent regulation of mRNA by TDP-43.

Furthermore, we find that downregulation of intron-free *hParkin* requires the RRM1 and GRD domains of TDP-43 (Fig. 4), indicating that this regulation depends on both the RNA-binding and the protein–protein interaction of TDP-43. It is reasonable to speculate that TDP-43 may bind to the coding region of *Parkin* via the RRM1 motif and recruit other proteins that regulate mRNA stability via the GRD domain. Alternatively, TDP-43 might indirectly decrease *Parkin* mRNA levels by regulating other RNA-binding proteins. Lending support to this possibility, TDP-43 regulates the RNA splicing of the PUF-domain-containing protein PUM1, which regulates mRNA stability^38^. Also, we notice that the coding region of *Parkin* contains a TGTAAAGA sequence, which encodes the mRNA that is only one nucleotide different from the PUF-binding sequence (UGUANAUA) and might be recognized by PUM1^39^. However, as the PUF recognition sequence usually locates within 3′UTRs, the exact mechanism of this enigmatic intron/UTR-independent regulation of *Parkin* mRNA is yet to be unraveled by additional research in the future.

Although TDP-43 does not directly regulate *PINK1* mRNA or protein levels, we find that it selectively impedes PINK1 protein turnover and causes cleaved PINK1 to accumulate in the cytoplasm due to the impairment of proteasomal degradation (Fig. 5 and Fig. S4). This alteration may cause cytotoxicity at two levels—on one side, it may reduce PINK1 interaction with its normal mitochondrial substrates such as NdufA10^40^, leading to mitochondrial dysfunction; on the other side, the cytosolic accumulation of PINK1 may cause gain of toxicity due to ectopic or increased phosphorylation of cytosolic substrates such as Parkin, ubiquintin, and others yet to identify^41,42^. Along the line, although we did not observe substantial induction of mitophagy by TDP-43 OE in our systems (Fig. S7), it is reported that cytosolic accumulation of cleaved PINK1 induces non-selective mitophagy^43^ and constitutive activation of PINK1 triggers non-apoptotic cell death that is independent of mitophagy^44^. In this study, we show that the increase of cleaved PINK1 reduces the reserve respiration capacity of mitochondria (Fig. 6), which may contribute to disease progression in TDP-43 proteinopathy.

The misregulation of the Parkin–PINK1 pathway shall have profound detrimental consequences. LOF mutations of the *Parkin* and *PINK1* genes are associated with juvenile PD^19,20^. In flies, *Parkin* and *PINK1* LOF mutants exhibit mitophagy defects and cell death in muscles, which subsequently cause locomotion defects and reduce the longevity^45–48^. Moreover, mitochondrial fragmentation has been observed in transgenic mouse and fly models of TDP-43^33,34,49^ as well as fibroblasts obtained from patients carrying TDP-43 mutations^50^. Since the Parkin–PINK1 pathway also regulates mitochondrial fission-fusion proteins^51,52^, their misregulation may contribute to TDP-43-induced mitochondrial over-fission. Similarly, the substrates of Parkin range from proteins regulating mitochondrial dynamics, transport, components of the electron transport chain, to proteins functioning in the proteasome and the nucleus^53^. Therefore, the TDP-43-mediated downregulation of *Parkin* is likely to have comprehensive deleterious effects.

**Fig. 8:**
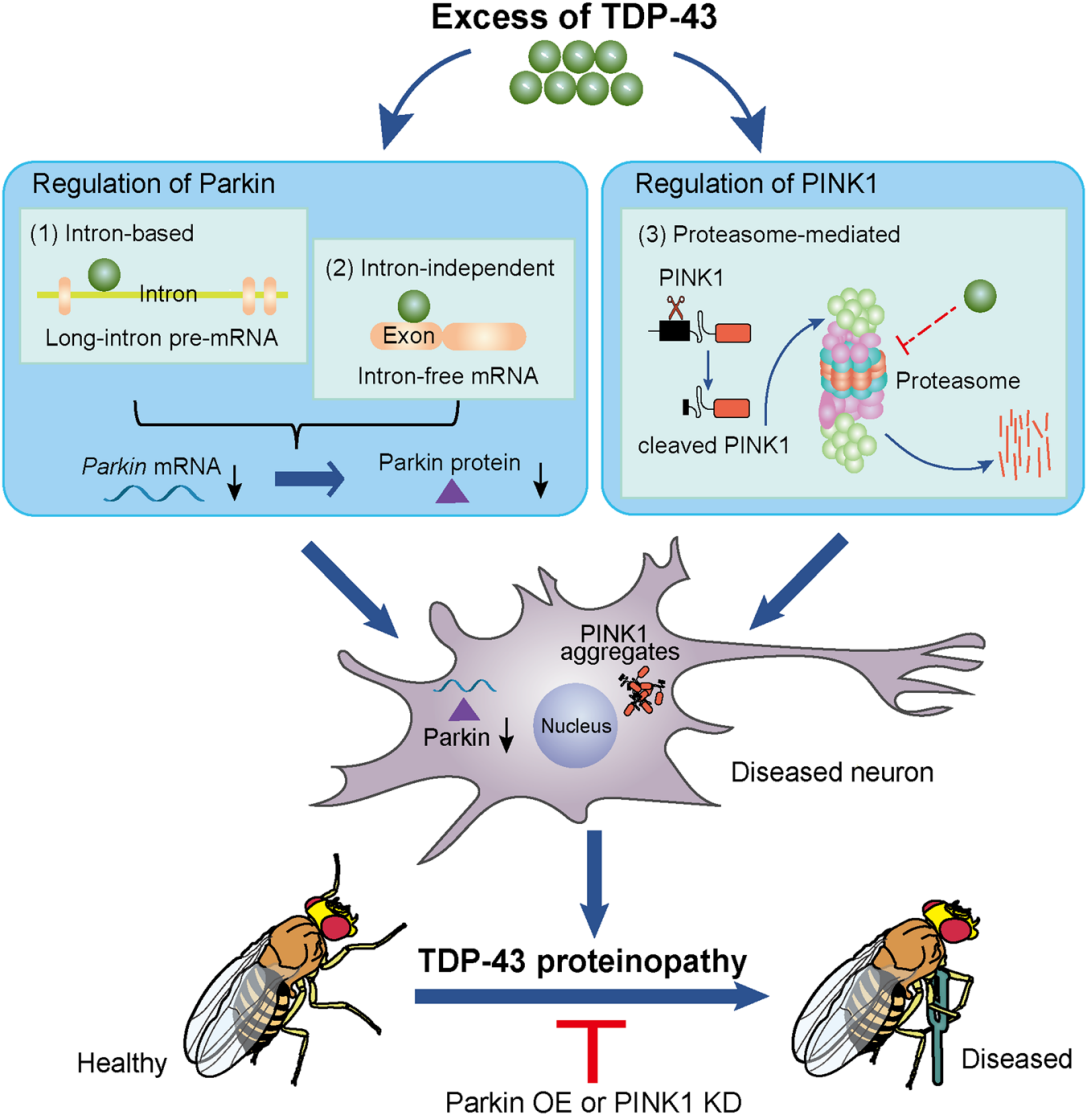
A schematic model of the multilevel misregulation of Parkin and PINK1 in TDP-43 proteinopathy. TDP-43 protein inclusion is a pathological hallmark of ALS and other neurodegenerative diseases of TDP-43 proteinopathies. In this study, we reveal that TDP-43 OE misregulates the mitochondrial quality control genes *Parkin* and *PINK1* differently and at multiple levels. In addition to (1) the previously reported TDP-43 function in regulating long intron-containing pre-mRNA of *Parkin*, we find that (2) *Parkin* mRNA without any intron can also be robustly decreased by TDP-43, suggesting an unidentified, intron-independent mechanism. The intron-based and the intron-independent regulations together lead to a significant reduction of Parkin protein abundance. Meanwhile, (3) excessive TDP-43 impairs proteasomal activity that impedes the turnover of cleaved PINK1, resulting in cytosolic accumulation of insoluble PINK1 aggregates. Consistent with the changes at the cellular level, *Parkin* OE but *PINK1* KD in a *Drosophila* model of TDP-43 significantly suppresses the degenerative phenotypes

In summary, in this study we find that *Parkin* and *PINK1* are differentially misregulated in TDP-43 proteinopathy (Fig. 8). TDP-43 OE reduces *Parkin* mRNA levels, which involves both intron-based and intron-independent mechanisms. In the meantime, excessive TDP-43 impairs the proteasomal activity, which hinders PINK1 turnover and causes cytosolic accumulation of cleaved PINK1. Consistently, upregulation of *Parkin*, whereas downregulation of *PINK1* suppresses TDP-43-induced neurodegeneration in a *Drosophila* model of ALS. Together, we propose that *Parkin* and *PINK1* are differentially misregulated at RNA and protein levels, which may contribute to the pathogenesis of TDP-43 proteinopathy. As functions of Parkin and PINK1 beyond regulating mitophagy or mitochondria are increasingly revealed, our findings strongly suggest that differential therapeutic strategies need to be developed when considering the Parkin–PINK1 pathway as a common target for treating ALS.

## Materials and methods

### Plasmids and constructs

To generate the pCAG-Flag-Parkin, pCAG-PINK1-V5, and pCAG-hTDP-43-HA plasmids, DNA fragments encoding human Parkin, PINK1, and TDP-43 were amplified from YFP-PARK2 (Addgene #23955^54^), pcDNA-DEST47 PINK1 C-GFP (Addgene #13316^55^), and pcDNA3.1-TDP-43-Myc plasmid (a gift from H-Y Hu) by PCR using primers containing the Flag, V5, or HA-tag sequence. The PCR products were then subcloned into a pCAG vector^56^ using the *Xho*I/*Eco*RI sites. The pDsRed2-mito vector was from Clontech Laboratories, Inc.

The expression constructs of truncated TDP-43 were generated by homologous recombination. Briefly, the DNA fragments of truncated hTDP-43-HA were amplified by PCR and inserted into the cloning vector using ClonExpress MultiS One Step Cloning Kit (Vazyme). The constructs were then subcloned into the pCAG expression vector as above. The PCR primers used were listed below:

ΔRRM1-F1: 5′-ATGGCCATGGAGGCCGAATTCATGTCTGAATATATT-3′

ΔRRM1-R1: 5′-CTCATCTTGGCTTTGGGATGTTTTCTGGACTGCTC-3′

ΔRRM1-F2: 5′-CAAAGCCAAGATGAG-3′

ΔRRM1-R2: 5′-GATGAGATGTTTTCTGGACTGCTC-3′

ΔRRM2-F1: 5′-ATGGCCATGGAGGCCGAATTCATGTCTGAATATATT-3′

ΔRRM2-R1: 5′-CTATTGCTATTGTGCTTAGGGCTTCTCAAAGGCTCATCTT-3′

ΔRRM2-F2: 5′-CCTAAGCACAATAGC-3′

ΔRRM2-R2: 5′-CATGTCTGGATCCCCGCGGCCGCCTACATTCCCCAGCCAGA-3′

ΔGRD-F: 5′-ATGGCCATGGAGGCCGAATTCATGTCTGAATATATT-3′

ΔGRD-R: 5′-CATGTCTGGATCCCCGCGGCCGCCTAACTTCTTTCTAACTGTCTATT-3′

To generate the pCAG-PINK1^Δ1-104^-V5 plasmid, the DNA fragments of truncated PINK1 were amplified from FL PINK1 by PCR and inserted into the pCAG vector as above. The PCR primers used were:

PINK1^Δ1-104^-F: 5′-CATCATTTTGGCAAAGAATTCCACCATGGGGCTAGGGCTGGGCCTC-3′

PINK1^Δ1-104^-V5-R: 5′-GCTCCCCGGGGGTACCTCGAGTTACGTAGAATCGAGACCGAGGAGAGGGTTAGGGATAGGCTTACCCAGGGCTGCCCTCCATGAG-3′.

All constructs were verified by sequencing to ensure the integrity of the cloned open reading frames.

### Cell cultures and transfection

293T cells were cultured in Dulbecco’s modified Eagle medium (Invitrogen) supplemented with 10% (v/v) fetal bovine serum (BioWest) and GlutaMAX^TM^ (Invitrogen) at 37 °C in 5% CO_2_. Transient transfection was performed using Lipofectamine™ 2000 (Invitrogen) in Opti-MEM (Invitrogen). Cells were transfected for 48 h before harvest. For proteasome inhibition assays, MG-132 was added into the medium 3 h before harvest at a final concentration of 50 nM.

### Lentivirus production and primary neuron culture

Lentivirus was prepared according to the established protocols. Briefly, lentivirus packaging was performed by co-transfecting FHsynPW-TDP-43-HA with VSVG and delta 8.9 with the ratio of 1:1.5:2 into 293FT cells cultured in Opti-MEM I medium using PolyJet™ reagent (SignaGen). Culture supernatant was collected at 48 h after transfection and passed through a 0.45-μm filter. Viral particles were concentrated from culture supernatants by Lenti-X™ Concentrator (Clontech). Viral pellets used for neuronal infection were resuspended in Neurobasal medium (Invitrogen).

Primary hippocampal neurons were isolated from C57BL/6 mouse hippocampus at embryonic day 14 (E14) and cultured in serum-free Neurobasal medium (Invitrogen) supplemented with 2% B27 (Life Technologies), GlutaMax, and penicillin-streptomycin (Invitrogen). At 14 days in vitro (DIV), neurons were infected with FHsynPW-TDP-43-HA for 5 days before extraction for RNA or protein. For TDP-43^Q331K^ mouse-derived culture, the cortical neurons of the transgenics or their littermates were isolated at E16–E18 and cultured as above. The RNA and protein levels of TDP-43^Q331K^ neurons were examined at 15 DIV.

### TDP-43 transgenic mice

The TDP-43^Q331K^-myc transgenic mice^24^ were derived from the B6N.Cg-Tg (Prnp-TARDBP*Q331K) 103Dwc/J line (Jackson Laboratory, #017933) and maintained by breeding to the C57BL/6N background mice purchased from Vital River Laboratories Co., Ltd. All animal experiments were carried out in compliance with the institutional guidelines on the scientific use of living animals at Interdisciplinary Research Center on Biology and Chemistry (IRCBC) of the Chinese Academy of Sciences (Protocol #: IRCBC-2016-016, IRCBC Animal Care and Use Committee).

### Drosophila strains

The following strains were obtained from the Bloomington *Drosophila* Stock Center: RNAi-*mCherry* (#35785, a control for in vivo RNAi knockdown); UAS-*lacZ* (#8529, a control for UAS transgene expression); *elav*GS (#43642); UAS-*PINK1* (#51648); and UAS-*Parkin* (#51651). The following strains were obtained from the Tsinghua Fly Center: RNAi-*PINK1* (#38262) and RNAi-*Parkin* (#38333). The transgenic fly strain of UAS-TDP-43 was generated by ΦC31 integrase-mediated, site-specific integration, and the attP landing site stock used was UAS-phi2b2a;VK5 (75B1).

Flies tested in this study were raised on standard cornmeal media and maintained at 25 °C and 60% relative humidity. For adult-onset, neuronal expression of the UAS or RNAi transgenes using the *elav*GS driver,^59^ flies were raised on regular fly food supplemented with 80 μg/ml RU486 (TCI).

### Generation of the *dParkin*-(HA) KI fly line

#### Construction of the pCR2-TOPO-2xHA-PBac-3xp3-eGFP vector

A 2xHA tag donor vector was generated from the pCR2-TOPO-3xp3-eGFP HR vector following the protocols on the flyCRISPR website. The 2xHA-stop codon-fused pBac 3′-terminal repeat fragments were created based on the pHD-2xHA-ScarlessDsRed vector by PCR reaction using the following primers: F1_5′-ACGTGCCAGACTATGCTTAACCCTAGAAAGATAATCATATTGTGACGTAC-3′, F2_5′-ATGTTCCAGATTACGCTGGATATCCATACGACGTGCCAGACTATGCTTAA-3′, F3_5′-gctagcctgacccgggGAAGACCTTACCCATACGATGTTCCAGATTACGCTGGA-3′ R_5′-aattagatcccctaggTAAAAGTTTTGTTACTTTATAGAAGAAATT-3′.

A 338-bp PCR product was generated and cloned into the *Nde*I/*Avr*II sites of pCR2-TOPO-3xp3-eGFP HR vector by In-Fusion HD Cloning Kit (Clontech) to get a transition vector. The pBac 5′-terminal repeat fragment was created from pHD-2xHA-ScarlessDsRed by PCR reaction using the following primers:

F_5′-cttatcgaatacgcgtGATATCTATAACAAGAAAATATATATATAA-3′ R_5′-ctcgagcgatcatatgCGTCTCCTTAACCCTAGAAAGATAGTCTGCGTAAAAT-3′.

A 352-bp PCR product was generated and cloned into the *Mlu*I/*Nde*I sites of the transition vector by In-Fusion HD Cloning Kit to get the pCR2-TOPO-2xHA-PBac-3xp3-eGFP vector.

#### Construction of the pCR2-TOPO-*dParkin*-2xHA-PBac-3xp3-eGFP HR donor vector

The L-arm of the *dParkin* flanking sequence without the stop codon was generated by PCR of the genomic DNA from the W^1118^ flies with the following primers:

F_ 5′-atccactagtgctagcGGATTTTGCGGCTCCATTGAATCTAATCAA-3′

R_5′-aacatcgtatgggtaGCCGAACCAGTGAGCTCCCATGCAGTCGCG-3′.

A 1032-bp PCR product was generated and cloned into the *Nhe*I/*Bbs*I sites of pCR2-TOPO-2xHA-PBac-3xp3-eGFP by In-Fusion HD Cloning Kit to get a Parkin-2xHA transition vector. The R-arm of *dParkin* flanking sequence was generated similarly using the following primers:

F_5′-tctttctagggttaaTCCTTCGTCATCGTTGTACGTCGGCCCGACGTGTCCTTTTTG-3′

R_5′-tagatgcatgctcgagAGCGGCAAACCGAAAGGAATCTAGAACGTG-3′.

A 1149-bp PCR product was generated and cloned into the *Bsm*bI/*Xho*I sites of *dParkin*-2xHA transition vector by In-Fusion HD Cloning Kit to get the complete pCR2-TOPO-*dParkin*-2xHA-PBac-3xp3-eGFP HR donor vector.

#### Construction of the pBFv-U6.3-*dParkin*-stop gRNA vector

The target sites of *dParkin* gene for CRISPR/Cas9 recognition were designed using the flyCRISPR Optimal Target Finder platform of flyCRISPR website. Two target sites close to the stop codon of the *dParkin* gene were found: gRNA-t1 site 5′-GAAGGATTAGCCGAACCAGTGGG-3′ and gRNA-t2 site 5′-TCGTTGTACGTCGGCCCGACGGG-3′. The target site fragments were generated by primer annealing and cloned into the *Bbs*I sites of pBFv-U6.3 vector (modified from pBFv-U6.2) to get the pBFv-U6.3-*dParkin*-stop gRNA-t1 and gRNA-t2 plasmids.

#### Embryo injection and transformant selection

The two pBFv-U6.3-*dParkin*-stop gRNA plasmids were mixed with the pCR2-TOPO-*dParkin*-2xHA-PBac-3xp3-eGFP HR donor vector for nanos-Cas9 founder line injection. Following green fluorescent protein (GFP)-mediated identification of successful KI flies, the GFP marker was removed through a single cross to PBac transposase. The embryo injection and selection of correct transformants were performed by BestGene Inc. and confirmed by PCR in the lab.

### RNA extraction and real-time quantitative PCR

For quantitative PCR (qPCR), total RNA was isolated from fly heads, cell cultures, or mouse brain tissues using TRIzol (Invitrogen) according to the manufacturer’s instruction. After DNase (Promega) treatment to remove genomic DNA, the RT reactions were performed using All-in-One cDNA Synthesis SuperMix kit (Bimake). The cDNA was then used either for semiquantitative RT-PCR experiments by PCR amplification using the Taq Plus Master Mix (Vazyme) or real-time qPCR using the SYBR Green qPCR Master Mix (Bimake) with the QuantStudio™ 6 Flex Real-Time PCR system (Life Technologies). The mRNA levels of *actin* or *GAPDH* were used as an internal control to normalize the mRNA levels of genes of interest. The qPCR primers used in this study are listed below:

*hβ-actin* forward: 5′-GTTACAGGAAGTCCCTTGCCATCC-3′

*hβ-actin* reverse: 5′-CACCTCCCCTGTGTGGACTTGGG-3′

*hPINK1* forward: 5′-CCCAAGCAACTAGCCCCTC-3′

*hPINK1* reverse: 5′-GGCAGCACATCAGGGTAGTC-3′

*hParkin* forward: 5′-GTGTTTGTCAGGTTCAACTCCA-3′

*hParkin* reverse: 5′-GAAAATCACACGCAACTGGTC-3′

*hTDP-43* forward: 5′-GGGAAATCTGGTGTATGTTGTCA-3′

*hTDP-43* reverse: 5′-GAAAATCACACGCAACTGGTC-3′

PINK1-V5 forward: 5′-CAGACGTGAGACAGTTGGTG-3′

PINK1-V5 reverse: 5′-GTAGAATCGAGACCGAGGAGAG-3′

Flag-Parkin forward: 5′-CAAGGATGACGACGATAAGT-3′

Flag-Parkin reverse: 5′-GCTGGAAGATGCTGGTGT-3′

*mGAPDH* forward: 5′-CACCATCTTCCAGGAGCGAG-3′

*mGAPDH* reverse: 5′-CCTTCTCCATGGTGGTGAAGAC-3′

*mPINK1* forward: 5′-CACACTGTTCCTCGTTATGAAGA-3′

*mPINK1* reverse: 5′-CTTGAGATCCCGATGGGCAAT-3′

*mParkin* forward: 5′-TCTTCCAGTGTAACCACCGTC-3′

*mParkin* reverse: 5′-TCTTCCAGTGTAACCACCGTC-3′

*dActin* forward: 5′-GAGCGCGGTTACTCTTTCAC-3′

*dActin* reverse: 5′-GCCATCTCCTGCTCAAAGTC-3′

*dPINK1* forward: 5′-GAGCAACAGCAGCATCAGAA-3′

*dPINK1* reverse: 5′-TGATGTTTGAATTCGCTGGA-3′

*dParkin* forward: 5′-AGCGATGCCACGACAATAGAGC-3′

*dParkin* reverse: 5′-GCGAAGGTTCCTCCTCCTCCAA-3′

### Antibodies

The following antibodies were used for western blotting, immunoprecipitation, and immunofluorescence assays: mouse anti-FLAG (Sigma-Aldrich, F3165); mouse anti-HA (Proteintech, 66006-1); rabbit anti-HA (CST, 3724); rabbit anti-TDP-43 (Proteintech, 10782-2-AP); rabbit anti-PINK1 (Novus Biologicals, BC100-494); mouse anti-Parkin (Santa Cruz, sc-32282); mouse anti-V5 (Proteintech, 66007-1); mouse anti-GAPDH (Proteintech, 60004-1); rabbit anti-Tubulin (MBL, PM054); mouse anti-Lamin C (DSHB, LC28.26); and chicken anti-MAP2 (Abcam, ab5392). Horseradish peroxidase-conjugated secondary antibodies: anti-mouse (Sigma-Aldrich, A4416) and anti-rabbit (Sigma-Aldrich, A9169). Fluorescent secondary antibodies: anti-mouse Cy5 (Life Technologies, A10524), anti-rabbit Alexa Fluor^®^ 488 (Life Technologies, A11012), and anti-chicken Alexa Fluor^®^ 633 (Sigma-Aldrich, A-21103).

### Protein extraction

Fly heads or cultured cells were lysed in 2% SDS lysis buffer (100 mM Tris-HCl at pH 6.8, 2% SDS, 40% glycerol, 10% β-mercaptoethanol, and 0.04% bromophenol blue) containing protease and phosphatase inhibitor cocktails (Roche). For separation of soluble and insoluble proteins, cells were lysed on ice using RIPA buffer (50 mM Tris at pH 8.0, 150 mM NaCl, 1% NP-40, 5 mM EDTA, 0.5% sodium deoxycholate, and 0.1% SDS) supplemented with protease and phosphatase inhibitors (Roche). Samples were sonicated and then centrifuged at 13 000 × *g* for 20 min at 4 °C. The resulting supernatant was used as the soluble fraction and the pellets containing insoluble fractions were dissolved in a 9 M urea buffer (9 M urea, 50 mM Tris buffer, pH 8.0) after wash.

Fresh brains of deep-anesthetized mice were rapidly excised and the motor cortices were isolated and collected in sterile 1.5 ml micro-centrifuge tubes. The samples were quickly plunged into liquid nitrogen and stored at −80 °C until testing. The brain tissues were then lysed and homogenized in ice-cold RIPA buffer supplemented with protease and phosphatase inhibitors (Roche).

### Western blotting

Equivalent amounts of lysates were resolved by electrophoresis through a 10% Bis-Tris SDS-polyacrylamide gel electrophoresis gel (Invitrogen) and probed with the primary and secondary antibodies listed above. Detection was performed using the High-sig ECL Western Blotting Substrate (Tanon). Images were captured using an Amersham Imager 600 (GE Healthcare) and densitometry was measured using ImageQuant TL Software (GE Healthcare). The contrast and brightness were optimized equally using Adobe Photoshop CS6 (Adobe Systems Inc.). All experiments were normalized to GAPDH, tubulin, or actin levels as indicated in the blots and the values are plotted relative to the control (set to a value of 1) in across-assay comparison quantifications.

### Nuclear and cytoplasmic extraction

For nuclear–cytoplasmic fractionation, 20 flies per genotype were homogenized in the lysis buffer [50 mM Tris at pH 7.4, 10 mM NaCl, 0.5% NP-40, 0.25% Triton X-100, 1 mM EDTA, and protease inhibitors (Roche)] by incubating for 5 min on ice as reported^15^ followed by centrifuge at 3000 × *g* for 5 min at 4 °C. The supernatant was collected as the cytoplasmic fraction and the pellet was dissolved in tissue lysis buffer (Invitrogen) as the nuclear fraction.

### Immunocytochemistry and confocal imaging

293T cells or primary neurons were grown on Nunc Chambered Coverglasses (Lab-Tek) and transfected with the indicated plasmids for 24 h. Thereafter, cells were fixed in 4% paraformaldehyde in phosphate-buffered saline (PBS) for 15 min at room temperature, permeabilized with 0.5% Triton X-100 in PBS for 15 min, and blocked with 3% goat serum in PBST (PBS + 0.1% Triton X-100) for 1 h at room temperature. The above primary and secondary antibodies in the blocking buffer were then incubated at 4 °C overnight or at room temperature for 1 h. After three washes with PBST, cells were mounted on glass slides using Vectashield Antifade Mounting Medium with DAPI (Vector Laboratories).

Fluorescent images were taken with Leica TCS SP8 confocal microscopy system using a ×63 oil objective (numerical aperture = 1.4). Co-localization of Parkin or PINK1 with mitochondria was evaluated by the line scanning analysis of LAS X and the protein puncta were counted using the “Analyze Particles” module of ImageJ. Images were assembled into figures using Adobe Photoshop CS6.

### Proteasomal activity assay

Proteasomal activity was measured using a Proteasome Activity Assay kit (Abcam, ab107921). 293T cells were lysed in PBS with 0.5% NP-40 on ice for 10 min. The supernatant was obtained by centrifuge at 13 000 × *g* for 10 min at 4 °C. The proteasomal activity of the supernatant was determined by assaying the cleavage of a fluorogenic peptide substrate Suc-LLVY-AMC according to the manufacturer’s instruction. The fluorescence intensity was measured after the substrate peptide was incubated with the cell lysates at 37 °C at the end of the assay (60 min) using a microplate reader (BioTek, Ex/Em = 350/440 nm).

### OCR measurement

Mitochondrial respiration functions were evaluated by measuring the OCRs using the Seahorse XFe96 Analyzer (Agilent) per the manufacturer’s instruction. Briefly, 24 h after transfection, 4 × 10^4^ 293T cells were seeded onto 96-well microplates pre-coated with poly-l-lysine. On the next day, the OCRs (pmol/min) of the cells in XF base medium containing 1 mM pyruvate, 2 mM glutamine, and 10 mM glucose (Sigma-Aldrich) were assayed with the Seahorse CF Cell Mito Stress Test following sequential additions of 1 μM oligomycin, 0.5 μM Carbonyl cyanide-4-(trifluoromethoxy)phenylhydrazone, and a combination of 0.5 μM antimycin A and 0.5 μM rotenone.

### Fly lifespan and climbing assays

For the lifespan experiment, 20 flies/vial, 5–8 vials/group were tested. Flies were transferred to fresh fly food every 3 days and the number of dead flies of each vial was recorded. Flies lost through escape or accidental death were excluded from the final analysis. The median lifespan was calculated as the mean of the medians of each vial belonging to the same group, whereas the “50% survival” shown on the survival curves was derived from compilation of all vials of the group. For the climbing assay, 20 flies were transferred into an empty polystyrene vial and gently tapped down to the bottom of the vial. The number of flies that climbed over a distance of 3 cm within 10 s was recorded. The test was repeated three times for each vial and 5 vials of each genotype were tested.^57,58^

### Statistical analysis

Unless otherwise noted, statistical significance in this study is determined by unpaired, two-tailed Student’s *t*-test at *p < 0.05, **p < 0.01, and ***p < 0.001. Error bars represent the standard error of the mean.

## Electronic supplementary material

Supporting Information

## References

[1] Kiernan MC (2011). Amyotrophic lateral sclerosis. Lancet.

[2] Ling SC, Polymenidou M, Cleveland DW (2013). Converging mechanisms in ALS and FTD: disrupted RNA and protein homeostasis. Neuron.

[3] Neumann M (2006). Ubiquitinated TDP-43 in frontotemporal lobar degeneration and amyotrophic lateral sclerosis. Science.

[4] Arai T (2006). TDP-43 is a component of ubiquitin-positive tau-negative inclusions in frontotemporal lobar degeneration and amyotrophic lateral sclerosis. Biochem. Biophys. Res. Commun..

[5] Higashi S (2007). Concurrence of TDP-43, tau and alpha-synuclein pathology in brains of Alzheimer’s disease and dementia with Lewy bodies. Brain Res..

[6] Arai T (2009). Phosphorylated TDP-43 in Alzheimer’s disease and dementia with Lewy bodies. Acta Neuropathol..

[7] Toyoshima Y, Takahashi H (2014). TDP-43 pathology in polyglutamine diseases: with reference to amyotrphic lateral sclerosis. Neuropathology.

[8] Chang XL, Tan MS, Tan L, Yu JT (2016). The role of TDP-43 in Alzheimer’s disease. Mol. Neurobiol..

[9] Chen-Plotkin AS, Lee VM, Trojanowski JQ (2010). TAR DNA-binding protein 43 in neurodegenerative disease. Nat. Rev. Neurol..

[10] Lagier-Tourenne C, Polymenidou M, Cleveland DW (2010). TDP-43 and FUS/TLS: emerging roles in RNA processing and neurodegeneration. Hum. Mol. Genet..

[11] Ratti A, Buratti E (2016). Physiological functions and pathobiology of TDP-43 and FUS/TLS proteins. J. Neurochem..

[12] Lagier-Tourenne C (2012). Divergent roles of ALS-linked proteins FUS/TLS and TDP-43 intersect in processing long pre-mRNAs. Nat. Neurosci..

[13] Polymenidou M (2011). Long pre-mRNA depletion and RNA missplicing contribute to neuronal vulnerability from loss of TDP-43. Nat. Neurosci..

[14] Rot G (2017). High-resolution RNA maps suggest common principles of splicing and polyadenylation regulation by TDP-43. Cell Rep..

[15] Tollervey JR (2011). Characterizing the RNA targets and position-dependent splicing regulation by TDP-43. Nat. Neurosci..

[16] Deas E (2011). PINK1 cleavage at position A103 by the mitochondrial protease PARL. Hum. Mol. Genet..

[17] Yamano K, Youle RJ (2013). PINK1 is degraded through the N-end rule pathway. Autophagy.

[18] Pickrell AM, Youle RJ (2015). The roles of PINK1, parkin, and mitochondrial fidelity in Parkinson’s disease. Neuron.

[19] Kitada T (1998). Mutations in the parkin gene cause autosomal recessive juvenile parkinsonism. Nature.

[20] Silvestri L (2005). Mitochondrial import and enzymatic activity of PINK1 mutants associated to recessive parkinsonism. Hum. Mol. Genet..

[21] Cozzolino M, Ferri A, Valle C, Carri MT (2013). Mitochondria and ALS: implications from novel genes and pathways. Mol. Cell. Neurosci..

[22] Muyderman H, Chen T (2014). Mitochondrial dysfunction in amyotrophic lateral sclerosis—a valid pharmacological target?. Br. J. Pharmacol..

[23] Lee EB, Lee VM, Trojanowski JQ (2012). Gains or losses: molecular mechanisms of TDP43-mediated neurodegeneration. Nat. Rev. Neurosci..

[24] Arnold ES (2013). ALS-linked TDP-43 mutations produce aberrant RNA splicing and adult-onset motor neuron disease without aggregation or loss of nuclear TDP-43. Proc. Natl Acad. Sci. USA.

[25] Elden AC (2010). Ataxin-2 intermediate-length polyglutamine expansions are associated with increased risk for ALS. Nature.

[26] Kim HJ (2014). Therapeutic modulation of eIF2alpha phosphorylation rescues TDP-43 toxicity in amyotrophic lateral sclerosis disease models. Nat. Genet..

[27] Kondo S, Ueda R (2013). Highly improved gene targeting by germline-specific Cas9 expression in Drosophila. Genetics.

[28] Bassett AR, Tibbit C, Ponting CP, Liu JL (2013). Highly efficient targeted mutagenesis of *Drosophila* with the CRISPR/Cas9 system. Cell Rep..

[29] Narendra DP (2010). PINK1 is selectively stabilized on impaired mitochondria to activate Parkin. PLoS Biol..

[30] Cao X (2017). In vivo imaging reveals mitophagy independence in the maintenance of axonal mitochondria during normal aging. Aging Cell.

[31] Rana A, Rera M, Walker DW (2013). Parkin overexpression during aging reduces proteotoxicity, alters mitochondrial dynamics, and extends lifespan. Proc. Natl Acad. Sci. USA.

[32] Chen Y (2016). PINK1 and Parkin are genetic modifiers for FUS-induced neurodegeneration. Hum. Mol. Genet..

[33] Khalil B (2017). Enhancing Mitofusin/Marf ameliorates neuromuscular dysfunction in Drosophila models of TDP-43 proteinopathies. Neurobiol. Aging.

[34] Wang W (2013). The ALS disease-associated mutant TDP-43 impairs mitochondrial dynamics and function in motor neurons. Hum. Mol. Genet..

[35] Xu YF (2010). Wild-type human TDP-43 expression causes TDP-43 phosphorylation, mitochondrial aggregation, motor deficits, and early mortality in transgenic mice. J. Neurosci..

[36] Yung C, Sha D, Li L, Chin LS (2016). Parkin protects against misfolded SOD1 toxicity by promoting its aggresome formation and autophagic clearance. Mol. Neurobiol..

[37] Hebron M, Chen W, Miessau MJ, Lonskaya I, Moussa CE (2014). Parkin reverses TDP-43-induced cell death and failure of amino acid homeostasis. J. Neurochem..

[38] Xiao S (2011). RNA targets of TDP-43 identified by UV-CLIP are deregulated in ALS. Mol. Cell. Neurosci..

[39] Gerstberger S, Hafner M, Ascano M, Tuschl T (2014). Evolutionary conservation and expression of human RNA-binding proteinsand their role in human genetic disease. Adv. Exp. Med. Biol..

[40] Morais VA (2014). PINK1 loss-of-function mutations affect mitochondrial complex I activity via NdufA10 ubiquinone uncoupling. Science.

[41] Kane LA (2014). PINK1 phosphorylates ubiquitin to activate Parkin E3 ubiquitin ligase activity. J. Cell Biol..

[42] Koyano F (2014). Ubiquitin is phosphorylated by PINK1 to activate parkin. Nature.

[43] Lim GG (2015). Cytosolic PTEN-induced putative kinase 1 is stabilized by the NF-kappaB pathway and promotes non-selective mitophagy. J. Biol. Chem..

[44] Akabane S (2016). Constitutive activation of PINK1 protein leads to proteasome-mediated and non-apoptotic cell death independently of mitochondrial autophagy. J. Biol. Chem..

[45] Clark IE (2006). *Drosophila* pink1 is required for mitochondrial function and interacts genetically with parkin. Nature.

[46] Greene JC (2003). Mitochondrial pathology and apoptotic muscle degeneration in Drosophila parkin mutants. Proc. Natl Acad. Sci. USA.

[47] Park J (2006). Mitochondrial dysfunction in Drosophila PINK1 mutants is complemented by parkin. Nature.

[48] Cha GH (2005). Parkin negatively regulates JNK pathway in the dopaminergic neurons of *Drosophila*. Proc. Natl Acad. Sci. USA.

[49] Magrane J, Cortez C, Gan WB, Manfredi G (2014). Abnormal mitochondrial transport and morphology are common pathological denominators in SOD1 and TDP43 ALS mouse models. Hum. Mol. Genet..

[50] Onesto E (2016). Gene-specific mitochondria dysfunctions in human TARDBP and C9ORF72 fibroblasts. Acta Neuropathol. Commun..

[51] Poole AC (2008). The PINK1/Parkin pathway regulates mitochondrial morphology. Proc. Natl Acad. Sci. USA.

[52] Yu W, Sun Y, Guo S, Lu B (2011). The PINK1/Parkin pathway regulates mitochondrial dynamics and function in mammalian hippocampal and dopaminergic neurons. Hum. Mol. Genet..

[53] Sarraf SA (2013). Landscape of the PARKIN-dependent ubiquitylome in response to mitochondrial depolarization. Nature.

[54] Narendra D, Tanaka A, Suen DF, Youle RJ (2008). Parkin is recruited selectively to impaired mitochondria and promotes their autophagy. J. Cell Biol..

[55] Beilina A (2005). Mutations in PTEN-induced putative kinase 1 associated with recessive parkinsonism have differential effects on protein stability. Proc. Natl Acad. Sci. USA.

[56] Chen Y (2014). Activity-induced Nr4a1 regulates spine density and distribution pattern of excitatory synapses in pyramidal neurons. Neuron.

[57] Hebron ML (2013). Parkin ubiquitinates Tar-DNA binding protein-43 (TDP-43) and promotes its cytosolic accumulation via interaction with histone deacetylase 6 (HDAC6). J. Biol. Chem..

[58] Wenqiang C (2014). Parkin-mediated reduction of nuclear and soluble TDP-43 reverses behavioral decline in symptomatic mice. Hum. Mol. Genet..

[59] Osterwalder T., Yoon K. S., White B. H., Keshishian H. (2001). A conditional tissue-specific transgene expression system using inducible GAL4. Proceedings of the National Academy of Sciences.

