# Supplementary material for: Distinct multilevel misregulations of Parkin and PINK1 revealed in cell and animal models of TDP-43 proteinopathy
Source: Cell Death Dis. 2018 Sep 20;9(10):953. doi: 10.1038/s41419-018-1022-y (PMC6148241; doi:10.1038/s41419-018-1022-y)
Supplement: Supplementary file 1 — Supporting Information [file 41419_2018_1022_MOESM1_ESM.docx]

**SUPPORTING Information**

**Distinct multi-level misregulations of Parkin and PINK1 revealed in cell and animal models of TDP-43 proteinopathy**

Xing Sun, Yongjia Duan, Caixia Qin, Jian-Chiuan Li, Gang Duan, Xue Deng, Jiangxia Ni, Xu Cao, Ke Xiang, Kuili Tian, Chun-Hong Chen, Ang Li, and Yanshan Fang


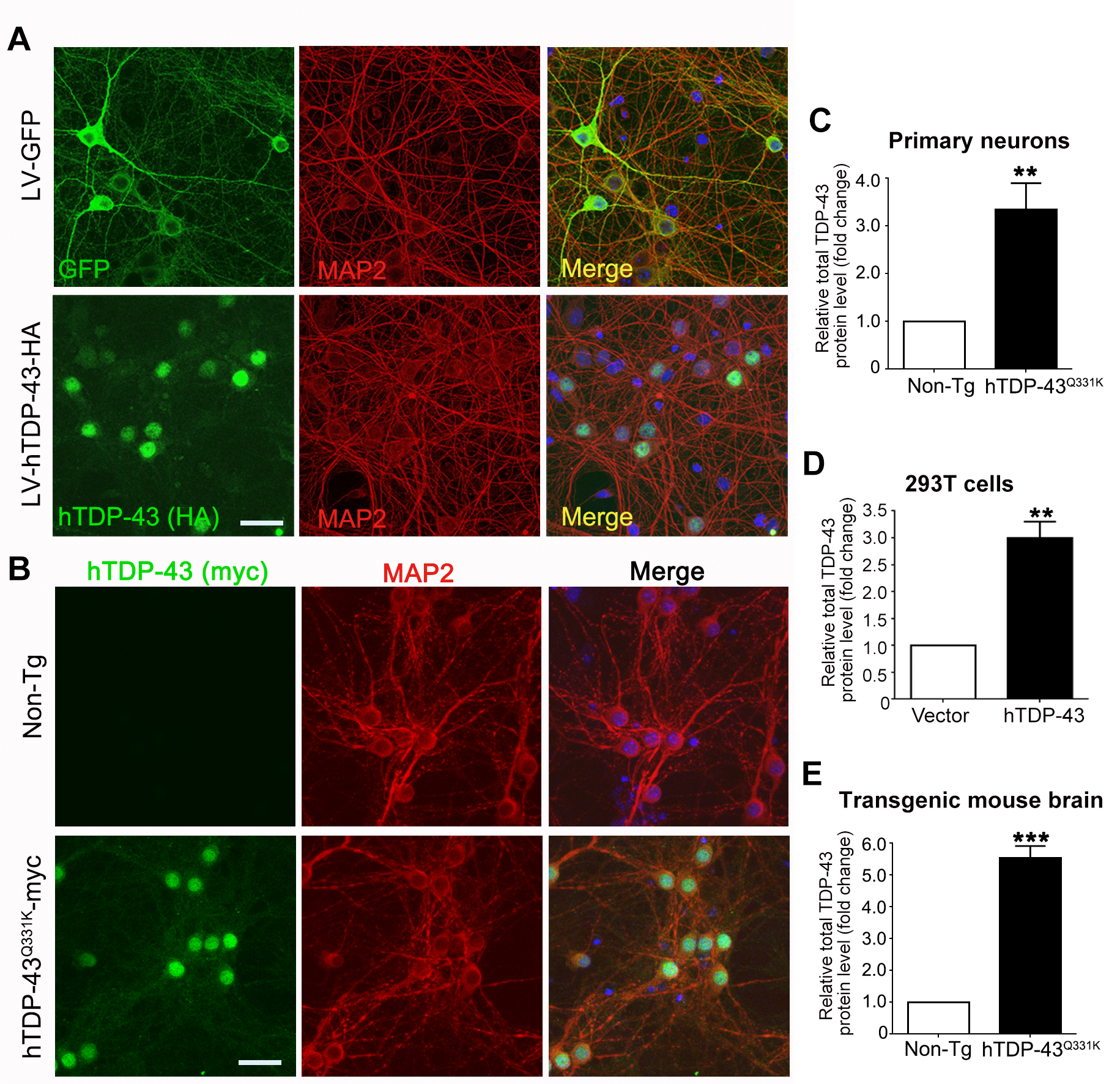
**Figure S1. Expression of hTDP-43 in lentivirus delivered and transgenic mouse-derived primary neurons.**

(**A**) The representative confocal images of mouse primary hippocampal neurons used in the qPCR analysis in Fig. 1B. The cultures are infected with the lentivirus (LV) to express either GFP (control) or hTDP-43-HA (green, stained for HA). (**B**) Representative images of cortical neurons derived from hTDP-43^Q331K^-myc mice, tested in Fig. 1C-1E. hTDP-43-myc (green), neuronal staining for MAP2 (red), and nuclei by DAPI (blue, in the merge images) are shown. Scale bars: 25 μm. As reported, both the wild-type hTDP-43 and the disease mutant hTDP-43^Q331K^ are predominantly localized in the nucleus of the mouse neurons (Arnold et al., 2013). (**C-E**) Overexpression efficiency of hTDP-43 in various models used in this study, including primary mouse neurons (C), 293T cells (D), and brains of transgenic mice (E), is evaluated by quantifying the relative levels of total TDP-43 proteins (the sum of the endogenous mouse or human TDP-43 and exogenously expressed hTDP-43) in the Western blotting analyses in Figure 1D, 3E and 3G, and 6A. The total protein level of TDP-43 in the control group of each experiment was set to 1. Data shown are means ± SEM, n = 3~5. Statistical significance is determined by Student’s *t*-test at ***p* < 0.01, ****p* < 0.001.

**
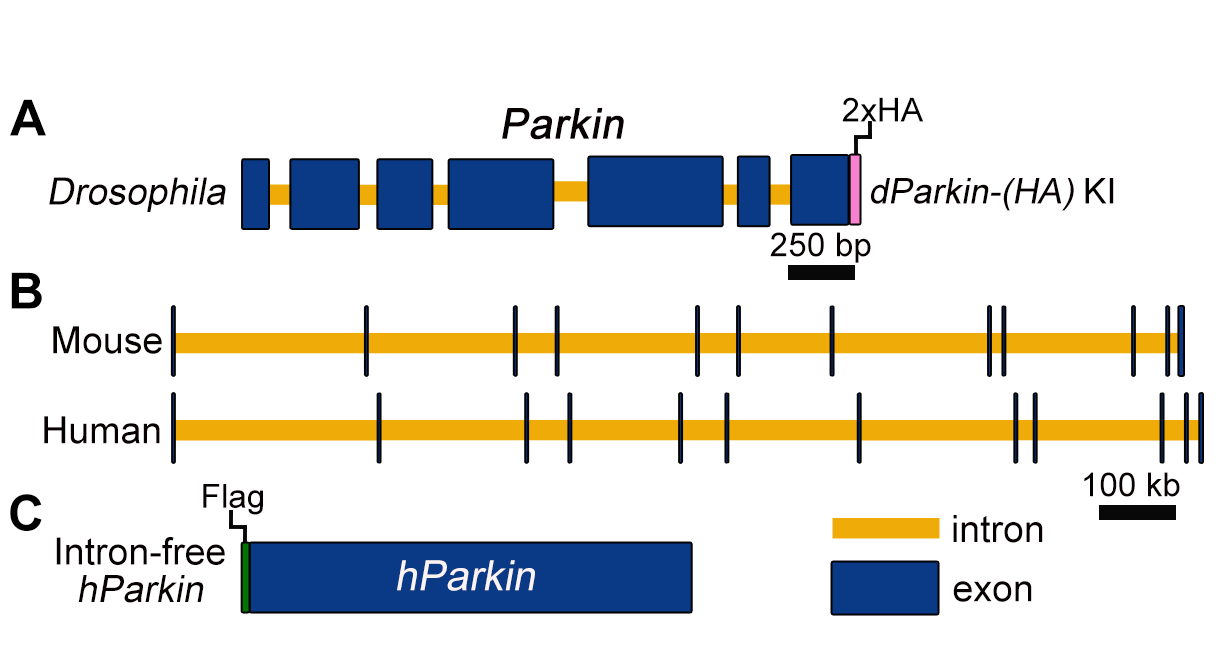
Figure S2. Schematic diagrams of the *Parkin* genes in *Drosophila*, mouse and human.**

(**A-B**) The schematic diagrams of the *Parkin* genes showing the exons (blue) and introns (yellow) in *Drosophila* (**A**) and mouse and human (**B**). In the *dParkin-(HA)* knock-in flies, a 2xHA tag is inserted into the fly genome at the C-terminus of the *dParkin* gene using CRISPR/Cas9-based gene editing as explained in Fig. 2B. Note the huge difference of the genetic scale bars between (A) and (B). The introns in the human and mouse *Parkin* genes are exceptionally long (most are > 100 kb), whereas the fly *Parkin* gene has only short introns (most are < 100 bp). (**C**) The constructed plasmid DNA for transient expression of human *Parkin* (*hParkin*) contains no intron (intron-free) but only the coding sequence and a Flag tag fused to the N-terminus.

**
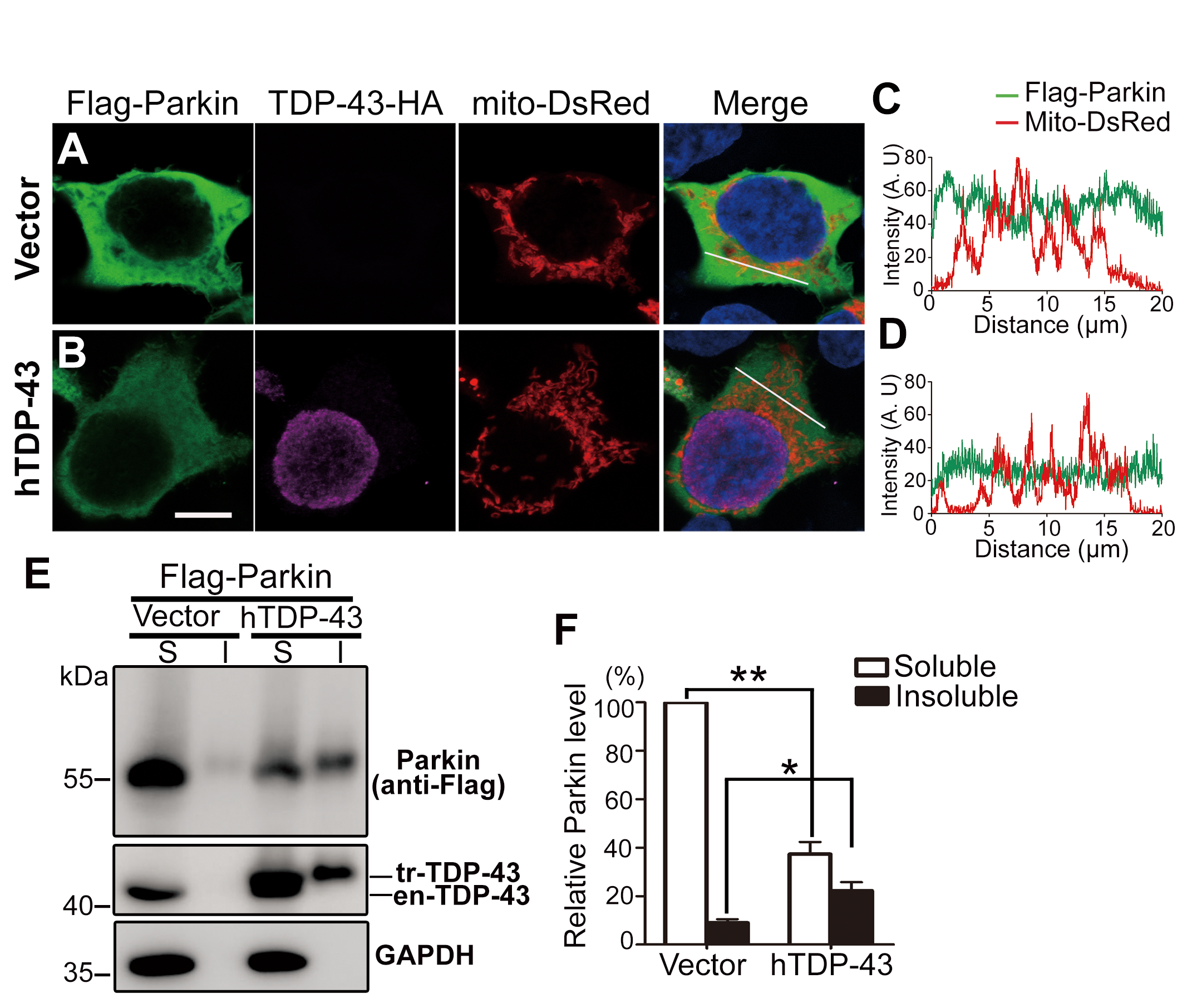
Figure S3. TDP-43 OE does not alter Parkin localization or form massive insoluble Parkin aggregates.**

(**A-B**) The representative confocal images show cells immunostained for Parkin (Flag) and TDP-43 (HA). Mitochondria are labeled by mito-DsRed (red), the nucleus is stained by DAPI (blue). Scale bar: 10 μm. (**C-D**) The co-localization of Parkin with mitochondria is evaluated by line scanning analysis. (**E-F**) Flag-Parkin is co-transfected with the empty vector (control) or hTDP-43-HA in 293T cells. The RIPA-soluble and insoluble (resolved in 9M urea) fractions of the cell lysates are analyzed by Western blotting in (**E**). S, soluble; I, insoluble; en-TDP-43, endogenous TDP-43; tr-TDP-43, transfected hTDP-43-HA. All protein levels are normalized to GAPDH and quantified in (**F**). The relative protein level of soluble Parkin in the control group is set to 100%. Data are shown as means ± SEM, *n* = 3. Statistical significance is determined by Student’s *t*-test at **p* < 0.05, ***p* < 0.01. hTDP-43 OE slightly increases insoluble Parkin but robustly decreases soluble and overall Parkin levels. Together, TDP-43 OE does not alter the subcellular distribution of Parkin or cause insoluble Parkin aggregates to form. This is in sharp contrast to the effect of TDP-43 on PINK1 protein solubility and subcellular localization in Fig. 5.


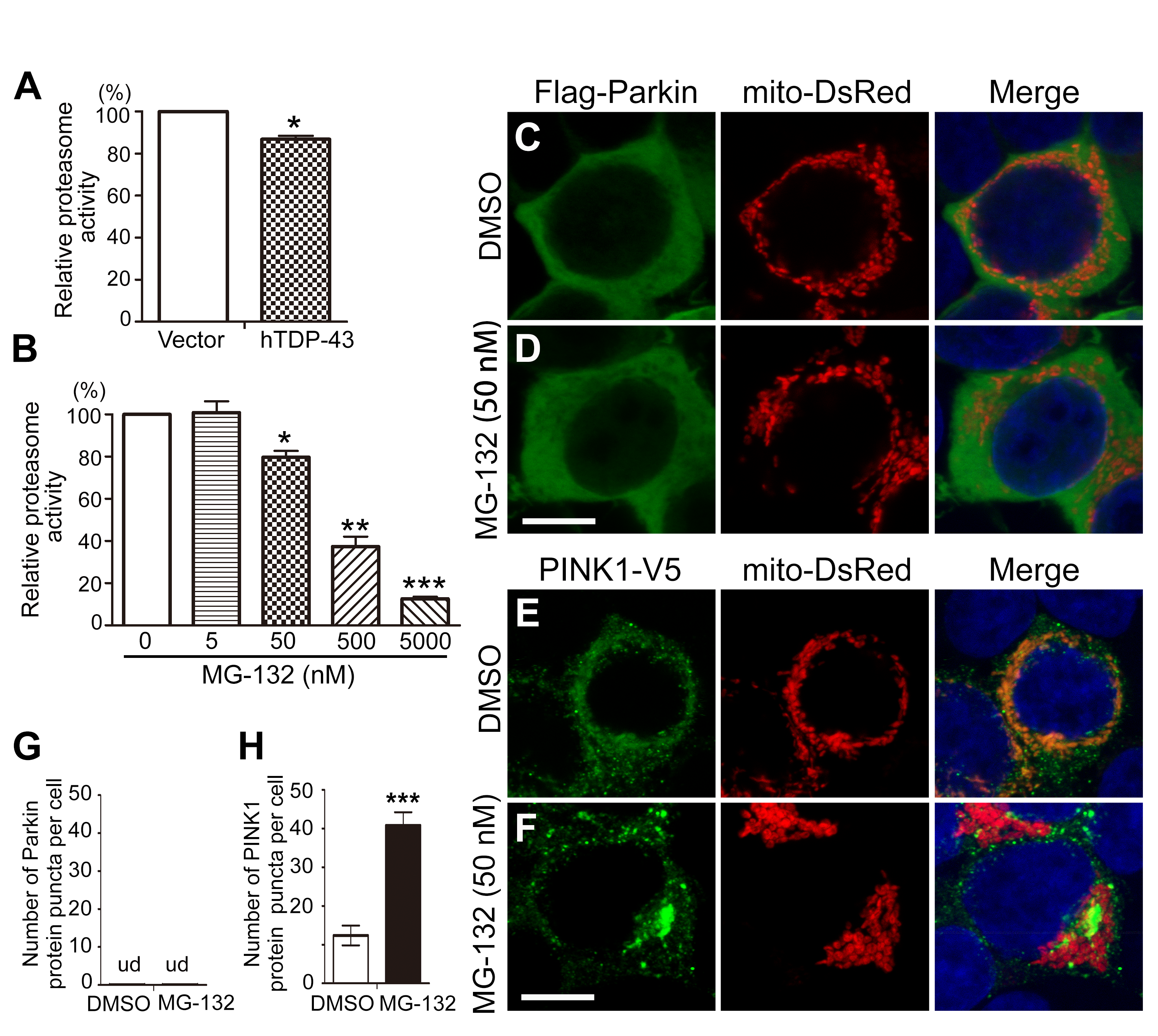
**Figure S4. Excessive TDP-43 moderately impairs proteasomal activity that selectively hinders PINK1 but not Parkin turnover.**

(**A**) The proteasome activity of the cells in the absence or presence of TDP-43 OE. (**B**) 293T cells treated with the proteasome inhibitor MG-132 at indicated concentrations for 3 h before lysed and the proteasomal activity is subsequently determined by an *in vitro* fluorogenic peptide cleavage assay. The relative proteolytic activities are shown as average percentages to the total fluorescence intensity of the control group at the end of the assay (set to 100%). The proteasomal activity of the cells transfected with hTDP-43-HA is ~15% lower than the control cells. (**C-F**) 293T cells transfected with Flag-Parkin (**C-D**) or PINK1-V5 (**E-F**) are treated with DMSO or 50 nM of MG-132 for 3 h. Cells are then fixed and immunostained for Parkin (Flag) or PINK1 (V5), and mitochondria are labeled by mito-DsRed. Merge shows all channels and the nucleus stained by DAPI in blue. Scale bars: 10 μm. (**G-H**) The average numbers of Parkin or PINK1 protein puncta per cell in (C-D) and (E-F) are counted and shown in (**G**) and (**H**), respectively. Data are means ± SEM of ~100 randomly selected cells per group, from pooled results of three independent repeats. Statistical significance is determined by Student’s *t*-test at **p* < 0.05, ***p* < 0.01, ****p* < 0.001; ud, undetected.

**
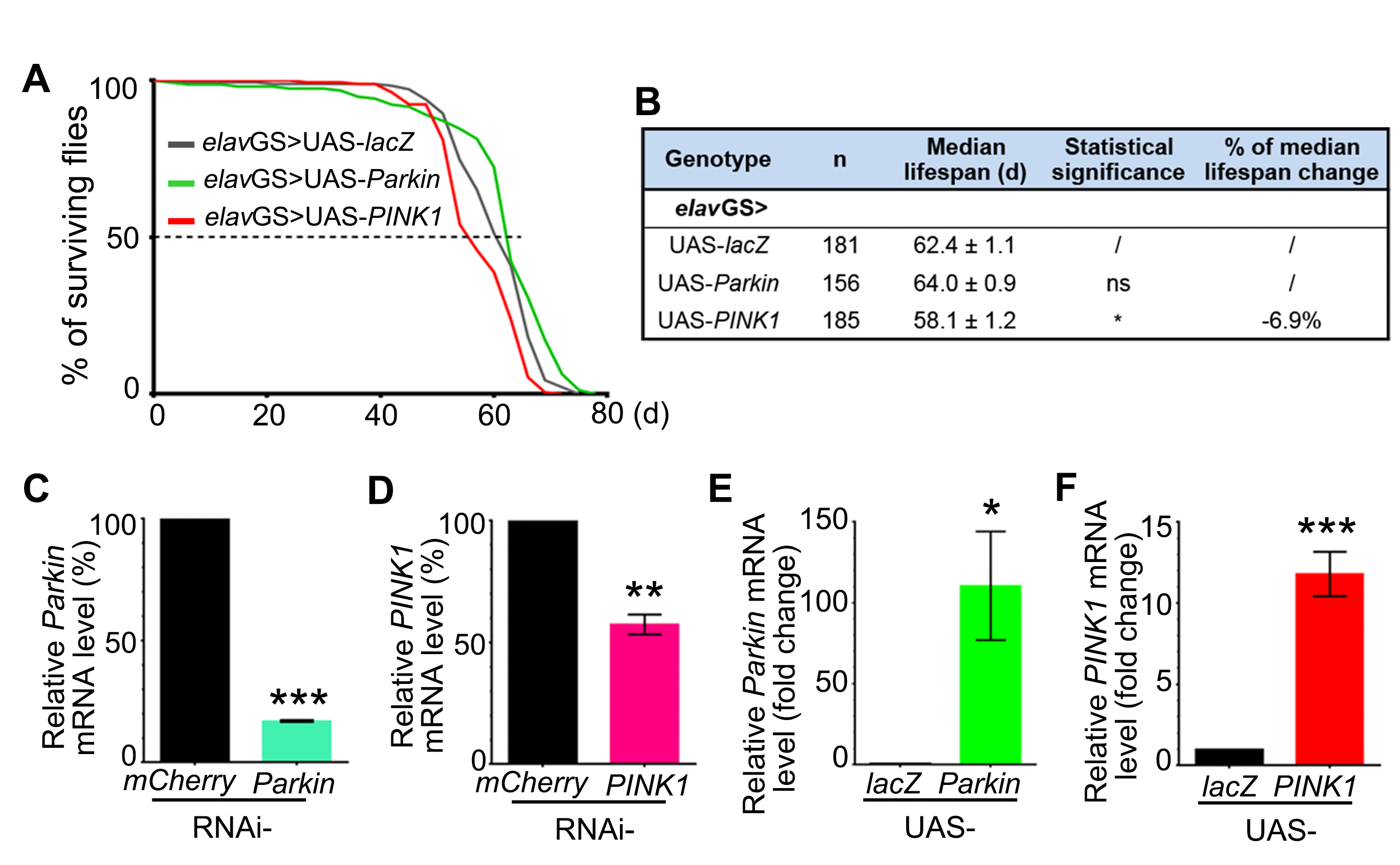
Figure S5. Transgenic KD or OE of *Parkin* and *PINK* in the fly neurons.**

(**A-B**) Neuronal OE of *Parkin* or *PINK1* in non-diseased flies (by the *elav*GS driver, induced on day 1 after adult flies eclosed from the pupa case) does not show a “general” beneficial effect to the longevity. Instead, *PINK1* OE in adult neurons significantly reduces the fly median lifespan by~6.9% compared to the control group (UAS-*lacZ*). (**C-F**) qPCR analyses of the KD efficiency or OE levels of transgenic RNAi-*Parkin* (**C**), RNAi-*PINK1* (**D**), *Parkin* OE (**E**), and *PINK1* OE (**F**) flies used in Fig. 7. *Parkin* or *PINK1* mRNA levels are normalized to *actin* mRNA (internal control), and the relative levels of *Parkin* and *PINK1* mRNA in the control groups (RNAi-*mCherry* or UAS-*lacZ* flies as indicated) are set to 100%. Data shown are means ± SEM, *n* = 3~5. Statistical significance is determined by Student’s *t*-test at **p* < 0.05, ***p* < 0.01, ****p* < 0.001; ns, not significant.


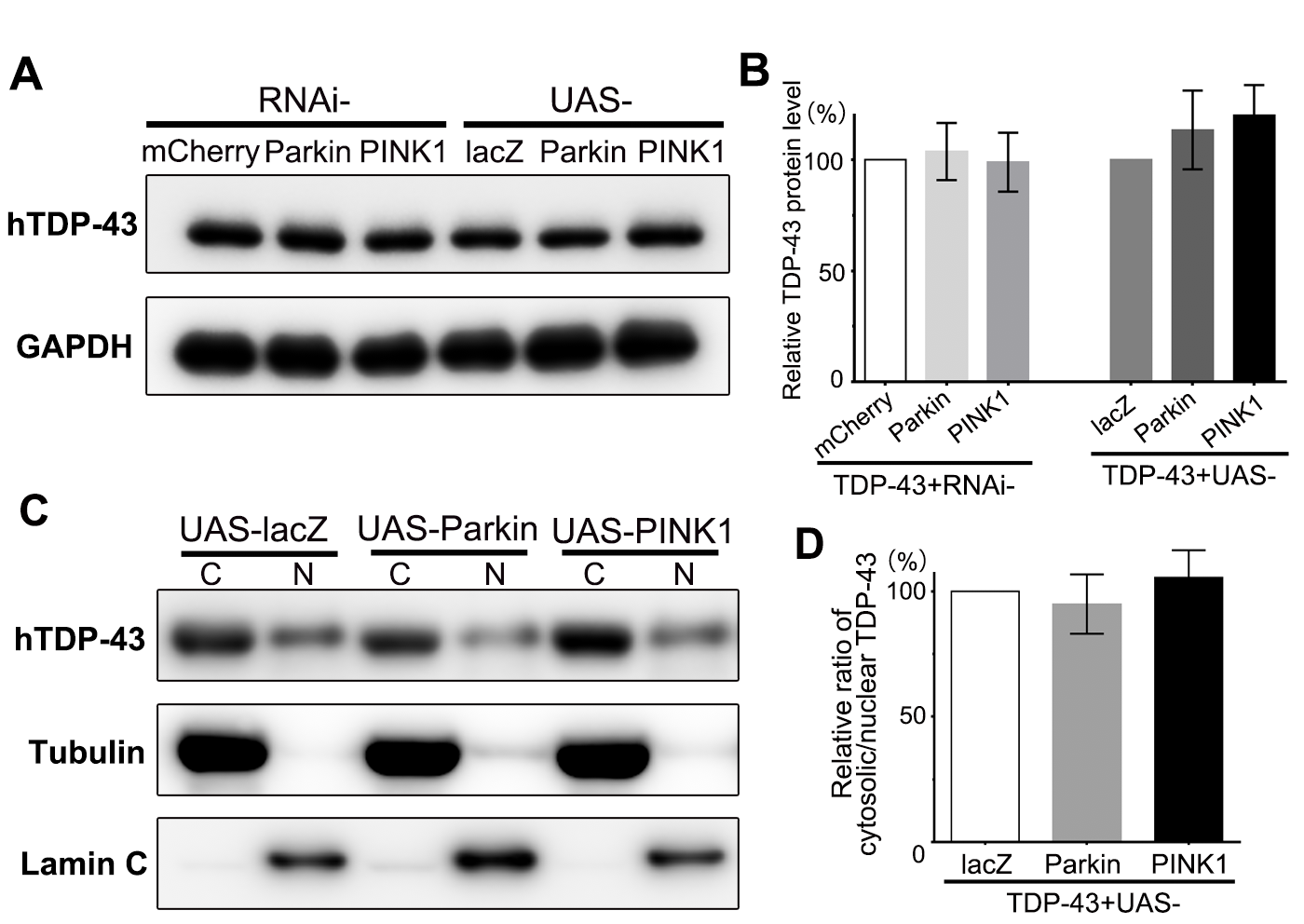
**Figure S6. Manipulating *Parkin* or *PINK1* levels in the fly head does not alter the abundance or subcellular distribution of hTDP-43 protein.**

(**A-B**) The effect of KD or OE of *Parkin* or *PINK1* on hTDP-43 protein levels in fly heads was examined by Western blot (**A**). TDP-43 protein levels are normalized to GAPDH and quantified in (**B**). (**C-D**) The fly heads of indicated genotypes are homogenized and lysed. The cytoplasmic and nuclear proteins are separated by fractionation and examined by Western blot (**C**). Tubulin and Lamin C are used as a cytoplasmic and a nuclear marker, respectively. C, cytosolic; N, nuclear. The cytosolic to nuclear (C/N) ratio of TDP-43 protein levels in the control group (UAS-*lacZ*) is set to 100% and the relative C/N ratios are quantified in (**D**). Data are means ± SEM, *n* = 3. Statistical significance is determined by Student’s *t*-test. No significant change of the protein abundacne or the subcellular distribution of TDP-43 is detected by down or up-regulation of *Parkin* or *PINK1* in adult fly neurons.


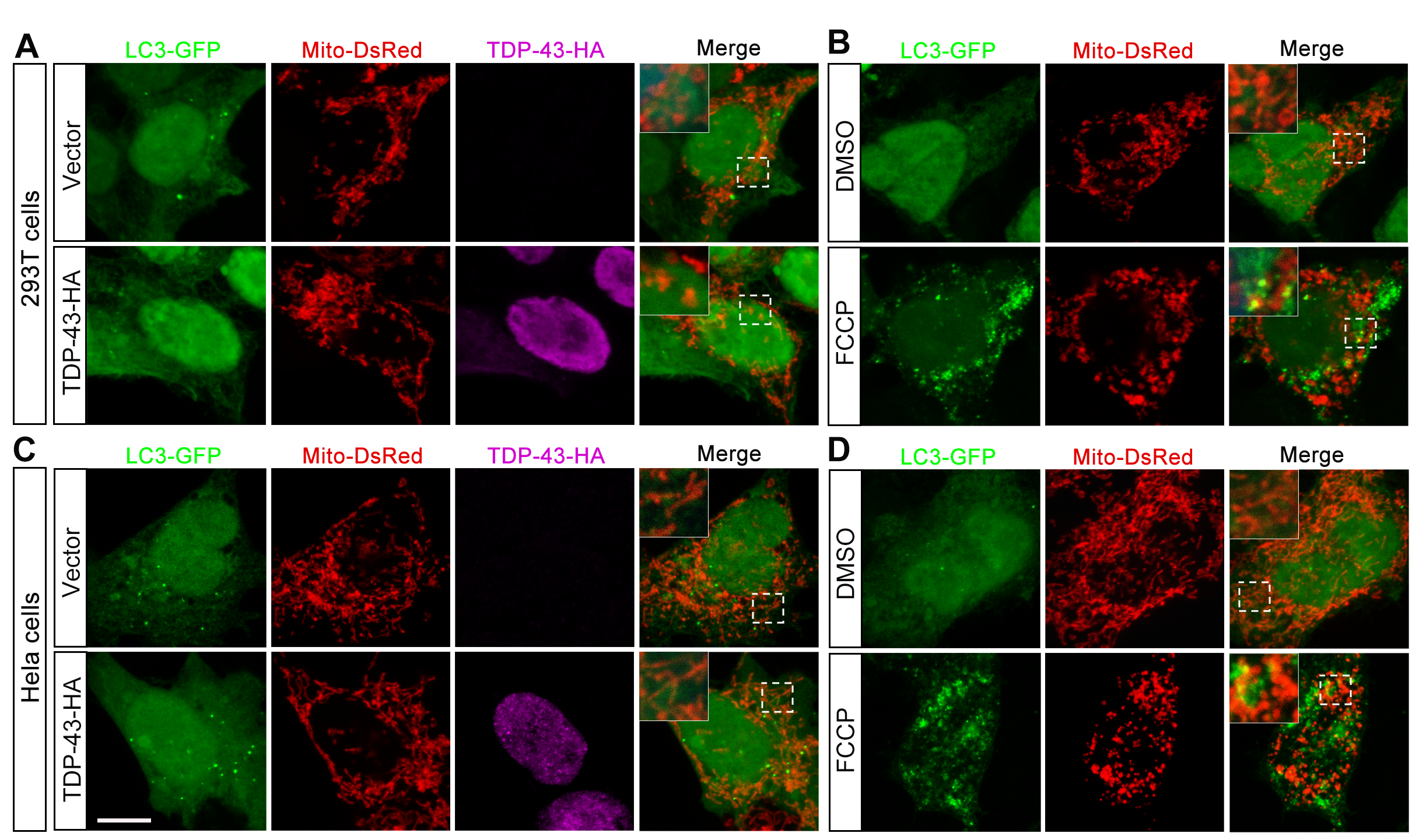
**Figure S7. TDP-43 OE does not enhance mitophagy in cells.**

(**A**) Representative confocal images of 293T cells overexpressing TDP-43-HA, which show normal mitochondria morphology and no induction of LC3-GFP puncta. (**B**) As a positive control, 293T cells treated with FCCP (20 μM, 1 h) show enhanced mitophagy – induction of LC3-GFP puncta and co-localization with mitochondria. (**C-D**) Since FCCP does not induce robust mitochondria fragmentation in 293T cells, we repeat the above experiment in HeLa cells, a more commonly used cell line for mitophagy study. Similarly, HeLa cells overexpressing TDP-43 do not show noticeable mitochondrial fragmentation or induction of LC3-GFP puncta (C); whereas as previously reported (Yamano and Youle, 2013), FCCP treatment (20 μM, 1 h) dramatically induces LC3-GFP puncta, mitochondrial fragmentation, and co-localization of them in HeLa cells (D). The levels of endogenous PINK1 in 293T cells and Parkin in HeLa cells are known to be low. To ensure reliable induction of mitophagy, all 293T cells are transfected with PINK1-V5 and all HeLa cells are transfected with Flag-Parkin in the above experiments. LC3-GFP is an autophagy marker; mito-DsRed labels mitochondria; expression of TDP-43-HA is confirmed by immunostaining with anti-HA antibody; and merge shows the LC3-GFP and mito-DsRed channels. The zoom-in inserts are shown for a closer examination of the co-localization of fragmented mitochondria with LC3-GFP puncta (mitophagy). Scale bar: 10 μm.
